# Explainable artificial intelligence incorporated with domain knowledge diagnosing early gastric neoplasms under white light endoscopy

**DOI:** 10.1038/s41746-023-00813-y

**Published:** 2023-04-12

**Authors:** Zehua Dong, Junxiao Wang, Yanxia Li, Yunchao Deng, Wei Zhou, Xiaoquan Zeng, Dexin Gong, Jun Liu, Jie Pan, Renduo Shang, Youming Xu, Ming Xu, Lihui Zhang, Mengjiao Zhang, Xiao Tao, Yijie Zhu, Hongliu Du, Zihua Lu, Liwen Yao, Lianlian Wu, Honggang Yu

**Affiliations:** 1 Renmin Hospital of Wuhan University, Wuhan, China; 2 Key Laboratory of Hubei Province for Digestive System Disease, Renmin Hospital of Wuhan University, Wuhan, China; 3 Hubei Provincial Clinical Research Center for Digestive Disease Minimally Invasive Incision, Renmin Hospital of Wuhan University, Wuhan, China; 4 Department of Gastroenterology, Wenzhou Central Hospital, Wenzhou, China; 5 Department of Gastroenterology, The Central Hospital of Wuhan, Tongji Medical College, Huazhong University of Science and Technology, Wuhan, China

**Keywords:** Gastric cancer, Experimental models of disease

## Abstract

White light endoscopy is the most pivotal tool for detecting early gastric neoplasms. Previous artificial intelligence (AI) systems were primarily unexplainable, affecting their clinical credibility and acceptability. We aimed to develop an explainable AI named ENDOANGEL-ED (explainable diagnosis) to solve this problem. A total of 4482 images and 296 videos with focal lesions from 3279 patients from eight hospitals were used for training, validating, and testing ENDOANGEL-ED. A traditional sole deep learning (DL) model was trained using the same dataset. The performance of ENDOANGEL-ED and sole DL was evaluated on six levels: internal and external images, internal and external videos, consecutive videos, and man–machine comparison with 77 endoscopists in videos. Furthermore, a multi-reader, multi-case study was conducted to evaluate the ENDOANGEL-ED’s effectiveness. A scale was used to compare the overall acceptance of endoscopists to traditional and explainable AI systems. The ENDOANGEL-ED showed high performance in the image and video tests. In man–machine comparison, the accuracy of ENDOANGEL-ED was significantly higher than that of all endoscopists in internal (81.10% vs. 70.61%, *p* < 0.001) and external videos (88.24% vs. 78.49%, *p* < 0.001). With ENDOANGEL-ED’s assistance, the accuracy of endoscopists significantly improved (70.61% vs. 79.63%, *p* < 0.001). Compared with the traditional AI, the explainable AI increased the endoscopists’ trust and acceptance (4.42 vs. 3.74, *p* < 0.001; 4.52 vs. 4.00, *p* < 0.001). In conclusion, we developed a real-time explainable AI that showed high performance, higher clinical credibility, and acceptance than traditional DL models and greatly improved the diagnostic ability of endoscopists.

## Introduction

Gastric cancer (GC) is the third cause of cancer-related mortality globally^1,2^. The prognosis of GC is highly related to the stage when diagnosed^3,4^. Early detection of GC is a cornerstone for effective treatment and prevention of mortality. White light endoscopy (WLE) is the first-line tool widely used to detect early gastric cancer (EGC)^5^. However, endoscopists have significant skill variations in detecting suspicious lesions, leading to a 20–40% missed diagnosis rate of EGC, which greatly threatens patients’ lives^6,7^. Therefore, it is a principle and of great value to enhance the diagnosis ability of EGC under WLE.

Deep learning (DL) has invoked tremendous progress in medical image analysis in recent years^8^. Several works have been conducted to achieve DL-based automatic diagnosis under WLE^9^. For instance, Tang et al. constructed an artificial intelligence (AI) system for EGC diagnosis under WLE, with a sensitivity of 85.9% in still images^10^. Our group previously developed an AI system (ENDOANGEL) to diagnose early gastric neoplasms under WLE with a sensitivity of 91.8%^11^. However, previous studies were mainly based on end-to-end DL algorithms, the diagnosis process of which is opacity and unexplainable “black box”^12^, difficult to be interpreted and understood by humans^13^, not to mention man–machine interaction that may help humans learn from AI or continuedly improve AI. This nature greatly affects the credibility and acceptability of AI systems in clinical practice.

Conversely, an explainable AI may increase the trust and acceptance of physicians and patients towards AI, reduce risks in healthcare, and is regulatory compliance of healthcare providers^14,15^. To achieve AI explainability in the medical field, many previous studies used a novel technique named LIME (Local Interpretable Model-Agnostic Explanations)^16,17^. Generally, they introduced understandable disturbance to the training data, for instance, blocking features in parts of an image, observing if it changes the answer of the AI, and finally determining the features contributing to the AI diagnosis. Other studies applied the Gradient-weighted Class Activation Mapping (Grad-CAM) technique to show AI explainability in visual^18,19^. Briefly, a heat map highlighting the most interested area of AI will be generated. It will help humans understand what features are being used to diagnose. However, the above methods were all post hoc ratiocination without exploring AI explainability, focusing on the model construction. In clinical applications, the internal decision-making logic of AI is still opaque, and doctors still cannot fully understand the diagnosis basis of AI. It is vital to construct an explainable AI system with the ability of man–machine interaction and a clear decision process.

In the present study, we proposed a novel method for developing AI systems based on feature-extraction and multi-feature-fitting and developed a real-time explainable AI system incorporated with domain knowledge named ENDOANGEL-ED (explainable diagnosis) using this method. ENDOANGEL-ED aimed to diagnose early gastric neoplasms (intraepithelial neoplasia, EGC, adenoma) with high performance.

## Results

### The retrospective datasets 1–5

Dataset 1, including 3612 images (1933 neoplastic images and 1679 non-neoplastic); dataset 2, including 433 images (115 neoplastic and 318 non-neoplastic); dataset 3, including 438 images (126 neoplastic and 312 non-neoplastic); dataset 4, including 115 videos clips timed 10.19 s on average (IQR, 9.00–12.00) containing 127 lesions (55 neoplasms and 72 non-neoplasms); dataset 5, including 85 videos clips timed 11.00 s on average (IQR, 12.00–13.56) containing 85 lesions (34 neoplasms and 51 non-neoplasms).

### The performance of feature-extraction models and fitting diagnosis models

The accuracy of feature-extraction models 1–6 for identifying spontaneous bleeding, protrusion, depression, boundary, surface, and tone reached 94.57%, 85.44%, 76.90%, 75.52%, 81.97%, and 81.31%, respectively, with semi-supervised algorithms performed better than supervised algorithms (Supplementary Table 1 and Supplementary Fig. 6–11). The other six-feature-extraction models were based on quantitative analysis. Their results were descriptive values, as shown in Supplementary Fig. 3.

**Fig. 1:**
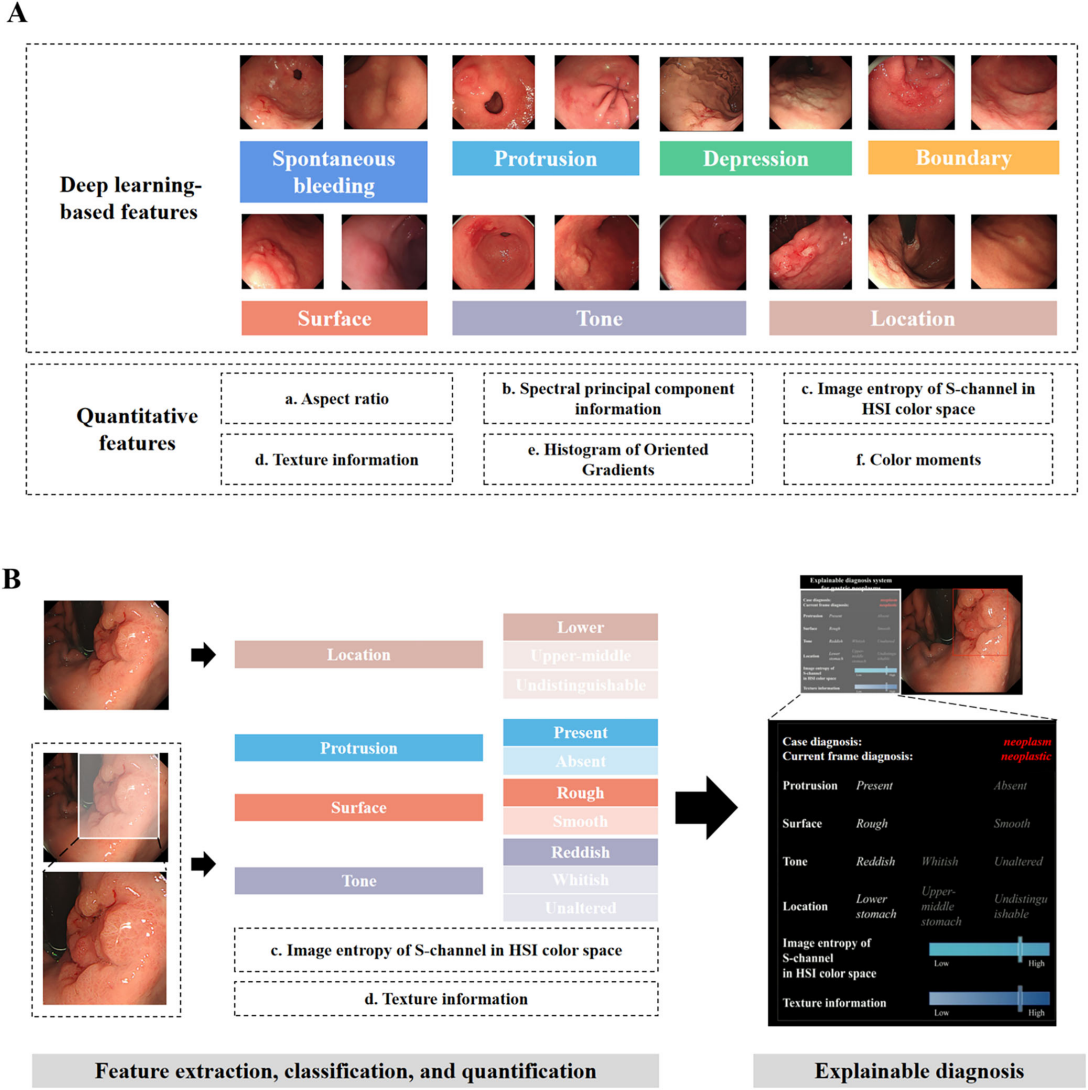
The schematic diagram of all feature indexes and the framework of developing ENDOANGEL-ED. **A** Thirteen features, including seven deep learning-based features and six quantitative features. **B** The framework of developing ENDOANGEL-ED. HIS (Hue, Saturation, Intensity).

The features extracted by the feature-extraction models were fed to seven ML-based fitting diagnosis models. The results showed that RF performed the best and was selected for constructing ENDOANGEL-ED. (Fig. 1A). The features selected by RF were surface, protrusion, tone, image entropy pf S-channel in HSI color space, Location, and Texture information, with a weight of 0.393, 0.190, 0.184, 0.092, 0.078, 0.063, respectively, which show the contributions of each feature on the final diagnosis. (Fig. 1B)

### The performance of ENDOANGEL-ED and sole DL in internal and external images

The best sole DL model was trained on different methods, and the best one was used to compare with ENDOENGEL-ED. (Fig. 1A) In the internal image test, the accuracy and specificity of ENDOANGEL-ED were better than that of the sole DL model (86.61% vs. 80.37%, *p* < 0.01; 87.11% vs. 76.73%, *p* < 0.001; McNemar test). In the external image test, the accuracy, specificity, and negative predictive value (NPV) of ENDOANGEL-ED were not significantly higher than the sole DL model (Table 1).

**Table 1:** The performance of ENDOANGEL-ED, sole DL model, and endoscopists in image and video tests.

	Accuracy (95% CI)	Sensitivity (95% CI)	Specificity (95% CI)	PPV (95% CI)	NPV (95% CI)
*Internal image test*					
ENDOANGEL-ED	86.61% (83.08–89.50%)	85.22% (77.60–90.56%)	87.11% (82.98–90.35%)	70.50% (62.45–77.45%)	94.22% (90.94–96.36%)
Sole DL model	80.37% (76.37–83.84%)**	90.43% (83.68–94.57%)	76.73% (71.78–81.04%)***	58.43% (51.09–65.42%)	95.69% (92.45–97.58%)
*External image test*					
ENDOANGEL-ED	77.17% (73.01–80.85%)	87.30% (80.36–92.03%)	73.08% (67.90–77.70%)	55.84% (48.86–62.60%)	93.44% (89.61–95.92%)
Sole DL model	74.89% (70.62–78.72%)	92.86% (86.99–96.20%)	67.63% (62.25–72.58%)	57.64% (50.76–64.23%)	96.17% (92.88–97.97%)
*Internal video test*					
ENDOANGEL-ED	81.10% (73.42–86.96%)	85.45% (73.83–92.44%)	77.78% (66.91–85.83%)	74.60% (62.66–83.72%)	87.50% (77.23–93.53%)
Sole DL model	71.65% (63.27–78.76%)	89.09% (78.17–94.90%)	58.33% (46.80–69.01%)*	62.03% (51.01–71.94%)	87.50% (75.30–94.14%)
*External video test*					
ENDOANGEL-ED	88.24% (79.69–93.49%)	97.06% (85.09–99.48%)	82.35% (69.74–90.43%)	78.57% (64.06–88.29%)	97.67% (87.93–99.59%)
Sole DL model	84.71% (75.58–90.84%)	94.12% (80.91–98.37%)	78.43% (65.37–87.51%)	74.42% (59.76–85.07%)	95.24% (84.21–98.69%)
All endoscopists (*n* = 46)	78.49 % (76.03–80.95%)***^^^	86.45% (84.22–88.67%)***^^^	73.19% (68.34–78.03%)**^	70.95% (67.20–74.70%)***^^	89.45% (88.18–90.73%)***^^^
Novices (*n* = 21)	78.77% (75.65–81.89%)***^^	85.58% (81.94–89.22%)***^^^	74.23% (67.97–80.49%)*^	70.96% (65.92–76.00%)**^	88.95% (86.85–91.06%)***^^^
Seniors (*n* = 14)	79.24% (75.17–83.31%)***	86.35% (81.90–90.79%)***^^	74.51% (66.09–82.94%)	71.56% (64.79–78.33%)	89.67% (87.13–92.21%)***^^
Experts (*n* = 11)	77.01% (68.85–85.16%)	88.24% (83.91–92.57%)***^	69.52% (54.15–84.90%)	70.16% (58.69–81.63%)	90.12% (87.62–92.63%)***^^
*Consecutive video test*					
ENDOANGEL-ED	79.76% (69.96–86.96%)	88.24% (65.67–96.71%)	77.61% (66.29–85.93%)	50.00% (33.15–66.85%)	96.30% (87.47–98.98%)
Sole DL model	70.24% (59.75–78.96%)	82.35% (58.97–93.81%)	67.16% (55.25–77.21%)	38.89% (24.79–55.14%)	93.75% (83.16–97.85%)

The McNemar test was used to compare the accuracy, sensitivity, and specificity between the ENDOANGEL-ED and the sole DL model. The *χ*^2^ test was used to compare the PPV and NPV between ENDOANGEL-ED and the sole DL model. Performance metrics between different levels of endoscopists and ENDOANGEL-ED and the sole DL model were compared using the Mann–Whitney U test.

*DL* deep learning, *CI* confidence interval, *PPV* positive predictive value, *NPV* negative predictive value.

*Significant difference between the target group and ENDOANGEL-ED. *, p < 0.05; **, *p* < 0.01; ***, *p* < 0.001.

^Significant difference between the target group and the sole DL. ^, *p* < 0.05; ^^, *p* < 0.01; ^^^, *p* < 0.001.

### The performance of ENDOANGEL-ED and sole DL in internal and external videos

**Fig. 2:**
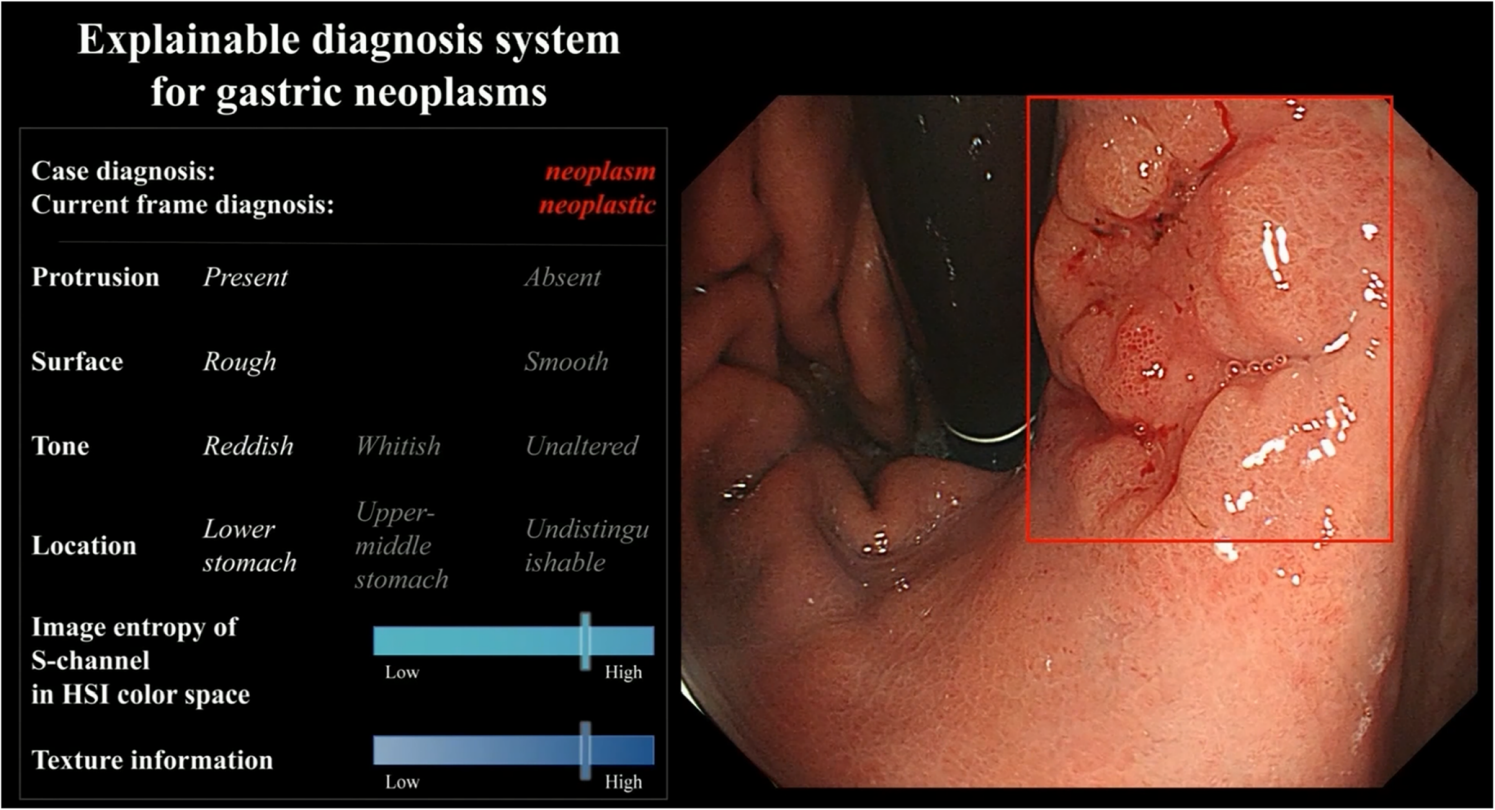
The system interface of ENDOANGEL-ED. The prediction of the six feature indexes and the diagnostic result were presented on the left.

In 127 internal videos, the specificity of ENDOANGEL-ED was significantly higher than that of the sole DL model (77.78% vs. 58.33%, *p* < 0.05; McNemar test). In 85 external videos, the accuracy, sensitivity, specificity, positive predictive value (PPV), and NPV of ENDOANGEL-ED were not significantly higher than the sole DL. The test result is presented in Table 1 (Fig. 2A, B).

### The performance of ENDOANGEL-ED and sole DL in consecutive videos

A total of 1441 patients who underwent EGD were consecutively enrolled. One thousand two hundred fifty-three patients who met the exclusion criteria were excluded. After lesion eligibility assessment, 84 lesions (17 neoplastic, 67 non-neoplastic) with pathology results from 82 patients were included in the analysis (Supplementary Fig. 12). All videos were edited into clips of 11.49 s per lesion (IQR, 10.00–14.00). The characteristics of patients and lesions are presented in Table 2. ENDOANGEL-ED’s accuracy, sensitivity, specificity, PPV, and NPV were not significantly higher than the sole DL model.

**Table 2:** Patient and lesion characteristics in consecutive videos.

Characteristics	Consecutive video test (*n* = 82)
Age, years (SD)	56.70 (11.80)
Sex, *n* (%)	
Male	50 (60.98)
Female	32 (39.02)
Recruitment, *n* (%)	
Inpatient	19 (23.17)
Outpatient	63 (76.83)
Neoplasms, *n*	
Gastric carcinoma	3
HGIN	7
LGIN	6
Tubular adenoma	1
Lymphoma	0
Non-neoplasms, *n*	
IM	21
Atrophy (without IM)	1
Chronic inflammation	43
Benign polyp	2
Total lesions	

*HGIN* high-grade intraepithelial neoplasia, *LGIN* low-grade intraepithelial neoplasia, *IM* intestinal metaplasia.

### Man–machine comparison

In the 127 internal videos, ENDOANGEL-ED showed significantly higher accuracy (81.10% vs. 70.61%, *p* < 0.001; Mann–Whitney U test), sensitivity (85.45% vs. 75.95%, *p* < 0.001; Mann–Whitney U test), specificity (77.78% vs. 66.44%, *p* < 0.001), PPV (74.60% vs. 65.29%, *p* < 0.001; Mann–Whitney U test), and NPV (87.50% vs. 78.20%, *p* < 0.001; Mann–Whitney U test) compared with all the 31 endoscopists. The accuracy, specificity, and PPV of ENDOANGEL-ED were comparable to that of the experts. The inter-rater agreement among endoscopists was 0.312 (fair agreement).

In 85 external videos, ENDOANGEL-ED had a significantly better performance in accuracy (88.24% vs. 78.77%, *p* < 0.001; Mann–Whitney U test), sensitivity (97.06% vs. 85.58%, *p* < 0.001; Mann–Whitney U test), specificity (82.35% vs. 74.23%, *p* < 0.05; Mann–Whitney U test), PPV (78.57% vs. 70.96%, *p* < 0.01; Mann–Whitney U test), and NPV (97.67% vs. 88.95%, *p* < 0.001; Mann–Whitney U test) compared with 21 novices. Compared to the 11 experts, the system was significantly better on sensitivity (97.06% vs. 88.24%, *p* < 0.001; Mann–Whitney U test) and NPV (97.67% vs. 90.12%, *p* < 0.001; Mann–Whitney U test). The inter-rater agreement between endoscopists was 0.504 (moderate agreement). The comparison results of internal and external videos are presented in Tables 1 and 3 (Fig. 2A, B).

**Table 3:** The performance of endoscopists with or without the ENDOANGEL-ED’s assistance.

	Accuracy (95% CI)	Sensitivity (95% CI)	Specificity (95% CI)	PPV (95% CI)	NPV (95% CI)
ENDOANGEL-ED	81.10% (73.42–86.96%)	85.45% (73.83–92.44%)	77.78% (66.91–85.83%)	74.60% (62.66–83.72%)	87.50% (77.23–93.53%)
Sole DL model	71.65% (63.27–78.76%)	89.09% (78.17–94.90%)	58.33% (46.80–69.01%)*	62.03% (51.01–71.94%)	87.50% (75.30–94.14%)
*Without AI assistance*					
All endoscopists (*n* = 31)	70.61% (67.59–73.63%)***^###^	75.95% (72.89–79.02%)***^^^^##^	66.44% (60.53–72.36%)***^^^^##^	65.29% (61.48–69.10%)***^^^###^	78.20% (75.88–80.51%)***^^^^###^
Novices (*n* = 21)	67.15% (63.68–70.63%)***^^###^	76.28% (72.64–79.92%)***^^^^##^	60.19% (53.48–66.89%)***^^###^	60.59% (56.93–64.24%)***^###^	76.64% (73.67–79.62%)***^^^^###^
Seniors (*n* = 7)	76.72% (74.62–78.81%)***^^^^###^	73.51% (63.38–83.64%)*^^##^	79.17% (68.47–89.86%)^^^##^	74.69% (67.00–82.37%)^^^##^	80.50% (75.65–85.34%)*^^#^
Experts (*n* = 3)	80.58% (70.92–90.23%)	79.39% (72.49–86.30%)*^^#^	80.56% (62.30–98.81%)^	76.25% (59.42–93.09%)^	83.69% (82.11–85.28%)*^^#^
*With AI assistance*					
All endoscopists (*n* = 31)	79.63% (77.40–81.86%)^^^	82.11% (79.24–84.98%)*^^^	77.73% (72.63–82.84%)*^^^	75.50% (72.39–78.61%)*^^^	85.56% (84.13–86.99%)
Novices (*n* = 21)	78.14% (75.13–81.31%)**^^^	84.15% (81.10–87.21%)^^^	73.55% (66.82–80.28%)^^^	72.34% (68.78–75.90%)^^^	86.32% (84.74–87.90%)
Seniors (*n* = 7)	82.23% (80.13–84.32%)^^^	75.06% (66.73–83.40%)**^^	87.70% (83.41–91.99%)**^^	82.88% (78.69–87.07%)***^^	82.53% (78.36–86.69%)*^
Experts (*n* = 3)	83.99% (71.57–96.41%)^	84.24% (81.65–86.84%)^	83.80% (63.88–100.00%)*^	80.40% (58.93–100.00%)*^	87.35% (83.09–91.61%)

The McNemar test was used to compare the accuracy, sensitivity, and specificity between the ENDOANGEL-ED and the sole DL model. The *χ*^2^ test was used to compare the PPV and NPV between ENDOANGEL-ED and the sole DL model. Performance metrics between different levels of endoscopists and ENDOANGEL-ED and the sole DL model were compared using the Mann–Whitney U test.

*DL* deep learning, *CI* confidence interval, *PPV* positive predictive value, *NPV* negative predictive value.

*Significant difference between the target group and ENDOANGEL-ED. *, *p* < 0.05; **, *p* < 0.01; ***, *p* < 0.001.

^Significant difference between the target group and the sole DL. ^, *p* < 0.05; ^^*, p* < 0.01; ^^^, *p* < 0.001.

^#^Significant difference between the AI-assisted and non-AI-assisted groups. ^#^*, p* < 0.05; ^##^, *p* < 0.01; ^###^, *p* < 0.001.

### MRMC study

With the assistance of ENDOANGEL-ED, the endoscopists’ accuracy (79.63% vs. 70.61%, *p* < 0.001; Mann–Whitney U test), sensitivity (82.11% vs. 75.95%, *p* < 0.01; Mann–Whitney U test), specificity (77.73% vs. 66.44%, *p* < 0.01; Mann–Whitney U test), PPV (75.50% vs. 65.29%, *p* < 0.001; Mann–Whitney U test), and NPV (85.56% vs. 78.20%, *p* < 0.001; Mann–Whitney U test) were significantly improved. Notably, the accuracy of novices was significantly lower than that of experts without ENDOANGEL-ED’s assistance (67.15% vs. 80.58%, *p* < 0.01; Mann–Whitney U test), while comparable to the experts after assistance (78.14% vs. 80.58%, *p* = 0.935; Mann–Whitney U test) (Table 3). The inter-rater agreement between endoscopists after AI assistance was 0.594 (moderate agreement). The diagnostic time with AI assistance was mildly shorter (52.28 vs. 58.68 min, *p* = 0.281; Mann–Whitney U test).

### Scale analysis

Thirty-one endoscopists completed the scale. The results showed that the explainable AI would increase the patient’s trust in the endoscopists, the endoscopists’ trust and acceptance of AI systems (4.35 vs. 3.90, *p* = 0.01; 4.42 vs. 3.74, *p* < 0.001; 4.52 vs. 4.00, *p* < 0.001; Mann–Whitney U test), compared with the traditional AI. Furthermore, the explainable AI would make endoscopists more interested and focus more on lesion observation and diagnosis (4.74 vs. 4.03, *p* < 0.001; 4.52 vs. 3.77, *p* < 0.001; Mann–Whitney U test), enhance the endoscopists’ confidence, and remind the endoscopists to think more comprehensively than traditional AI (4.71 vs. 4.06, *p* < 0.001; 4.71 vs. 4.00, *p* < 0.001; Mann–Whitney U test).

## Discussion

In this study, we developed a real-time explainable AI system named ENDOANGEL-ED incorporated with domain knowledge using feature-extraction and multi-feature-fitting methods. The ENDOANGEL-ED showed satisfactory results in diagnosing early gastric neoplasms in both image and video tests and performed better than the endoscopists. Furthermore, the system improved the diagnostic performance of endoscopists and was more acceptable and trusted than traditional AI.

In this study, based on thorough literature research and expert experience, we selected and extracted 13 features that were previously reported to be useful for diagnosis. By multi-feature-fitting using ML models, the RF model showed the best performance and included six features in diagnosis. The importance of these features was represented by weights (Fig. 1B). The top three important features were surface (rough or smooth), protrusion or not, and tone. These indicated that the abstract features proposed by previous studies could be subjectively analyzed and effectively integrated and obtain good diagnosis ability. This could provide new evidence for diagnosing neoplasm under WLE.

AI has been widely used in medical image analysis^20^. Generally, traditional DL only outputs a diagnosis conclusion without interpreting the decision process and diagnosis basis, which significantly impairs human trust and acceptance and causes severe social and ethical issues^21,22^. The USA and China’s official departments required AI systems to be open, explainable, and fair^23,24^. However, few studies in the medical field meet the requirements. Many studies used LIME and Grad-CAM techniques to show AI explainability logically and visually^16–19^. Nonetheless, they were both post hoc methods without exploring AI explainability during the model-constructing process.

Considering the importance of improving the explainability of the AI system, we tried to construct the ENDOANGEL-ED using novel methods in this study. However, diagnostic performance is the prerequisite for an AI model. Though an AI system has good explainability, it will be nonsense if the diagnostic performance is very poor. Theoretically, the DL models had powerful capabilities of feature-representation. However, they were questioned for their black-box nature and unexplainability^12,25,26^. Traditional ML models were not superior to DL models in feature-extraction and image analysis; nonetheless, their algorithm structures are visible and interpretable.

The primary purpose of this study is to construct high-performance explainable AI using novel methods. We believe that only with diagnostic performance not inferior to the traditional model, the advantage of the explainability nature of the AI could be discussed. Based on this, we proposed a novel method combining the strength of DL and ML models by feature-extraction (using DL models and quantitative analysis) and multi-feature-fitting (using ML models), and constructed an explainable AI system to diagnose early gastric neoplasms. The system gives not only prediction results but also the diagnostic basis to the operating endoscopists, greatly enhancing its transparency. Compared to traditional sole DL, explainable AI systems increased the patient’s trust in the endoscopists, the endoscopists’ trust, and the acceptance of AI systems. In addition, the explainable AI would make endoscopists more interested, focus more on lesion observation and diagnosis, and remind endoscopists to think more comprehensively than traditional AI. These indicated that the explainable AI might also have the potential to be useful in other scenarios, such as training programs.

Our method was similar to the learning process of humans. For diseases with obvious characteristics, humans could make a diagnosis by learning limited data. Generally, humans diagnose by learning typical pictures and summarizing their characteristics. Therefore, limited data on specific diseases may be of full use in the feature-fitting diagnosis process. We proved that explainable AI developed using this novel method showed high performance, though not significantly higher than the sole DL model constructed using the same training dataset. Therefore, our method may theoretically solve the long-standing problem of difficulty in collecting AI medical datasets and greatly promote the development and application of AI medical care.

This study has several limitations. First, only features related to the image were included in the analysis; clinical characteristics of the patients were not included. Adding clinic-related features in the algorithm may potentially further enhance the model. Further studies are needed to explore the performance of AI by combining the image and clinical feature indexes. Second, although the performance of the ENDOANGEL-ED was fully tested in internal, external, and consecutive videos and MRMC study, a real-time assessment of the model in clinical patients will further confirm its reliability and clinical validity and should be further conducted.

In conclusion, this study developed a real-time explainable AI system named ENDOANGEL-ED, incorporating domain knowledge, using feature-extraction and multi-feature-fitting methods. The system showed higher clinical credibility and acceptance than sole DL and greatly improved the diagnostic ability of endoscopists. The system can potentially improve the clinical safety and efficacy of AI systems in a real clinic.

## Methods

### Datasets

Five datasets were used for training, validation, and retrospective testing: (1) dataset 1, training and validation set; (2) dataset 2, internal image test set; (3) dataset 3, external image test set; (4) dataset 4, internal video test set; (5) dataset 5, external video test set. Datasets 1–4 were retrospectively collected from the Renmin Hospital of Wuhan University (RWHU) from November 2016 to November 2021. Dataset 3 was retrospectively collected from six hospitals, including Central Hospital of Wuhan, People’s Hospital of China Three Gorges University, Yichang Central People’s Hospital, Jingmen Petrochemical Hospital, Xiaogan Central Hospital, and Wenzhou Central Hospital from January 2019 to December 2019. Dataset 5 was retrospectively collected from the Beijing Cancer Hospital from June 9, 2020, to November 17, 2020.

The inclusion criterion of lesions: (1) focal lesions (only one focal lesion in the same sight of view). The exclusion criteria of lesions: (1) multiple lesions (more than one focal lesion in the same sight of view); (2) type I lesion, type III lesions, and ulcer; (3) the field of view was too close or too far; and (4) submucosal lesions. Images from the same lesion were not split between the training, validation, and test sets. The eligible images and ineligible images are shown in Supplementary Fig. 1. An expert endoscopist selected the images and videos according to the inclusion criteria of lesions. The internal and external videos were selected and edited by a research assistant under the guidance of an expert. The pathology results of the image and video test sets were reviewed by experienced gastroenterologists with over 10 years of experience in the pathological diagnosis of gastric abnormalities.

### Establishment of features

We determined the features related to gastric neoplasms through literature research. We searched by the keywords “white light endoscopy” OR “white light imaging”, “diagnosis” OR “feature” OR “characteristic”, “early gastric cancer” OR “gastric dysplasia” OR “gastric intraepithelial neoplasia” in the PubMed database between January 1, 2011, and December 31, 2021. A total of 164 pieces of literature were assessed. Furthermore, 149 records were excluded due to unrelated to gastric neoplasms diagnosis (*n* = 49), unrelated to WLE (*n* = 97), and case reports (*n* = 3). One of the 15 records was not retrieved because the full text was unavailable. Then, 14 records were assessed for eligibility; 8 were excluded due to unrelated to gastric neoplasms diagnosis features.

In addition, six records were added via manual search. Finally, 12 pieces of literature were included. Based on the literature, two expert endoscopists and two algorithmic engineers determined the features related to diagnosis. Ultimately, 13 features were selected for inclusion. The process of establishing features is shown in Supplementary Fig. 2.

### Construction of ENDOANGEL-ED

Thirteen features, including seven DL features and six quantitative features, were determined by literature research and included to construct the ENDOANGEL-ED.

Seven DL features were extracted using deep conventional neural networks (DCNN 1-7). Feature-extraction models 1–6 were trained, validated, and tested using images in Dataset 1. The images were not split among the training, validation, and testing sets. DCNN 1–6 were binary or three-category classification models aimed to determine the following six features, respectively: (1) spontaneous bleeding: whether a lesion has spontaneous bleeding; (2) protrusion: whether a lesion is protuberant or not; (3) depression: whether a lesion is depressed or not; (4) boundary: whether a lesion has a clear boundary; (5) surface: whether the surface of a lesion is rough or smooth; and (6) tone: whether the tone of a lesion is red, pale, or unaltered (the same tone as the background mucosa). We compared the performance of the supervised and semi-supervised algorithms in constructing DCNN 1-6. Before an image was sent to DCNN 1–6, it was first processed by our previously constructed YOLO-v3 model to localize the abnormities^27^. Briefly, YOLO-v3 was trained for detecting gastric lesions using 21,000 gastric images^11^ and could detect focal lesions with a sensitivity of 96.90%.

The seventh feature-extraction model was previously developed using the ResNet-50 algorithm for classifying 26 anatomical landmarks in esophagogastroduodenoscopy^28,29^. The location of a lesion was further classified into three categories, the upper-middle stomach, the lower stomach, and undistinguishable.

The quantitative features were extracted and analyzed based on the localized area by YOLO-v3. These quantitative features included:

1. The aspect ratio of the lesion area: the ratio of the width to the height of the lesion in an image, describing the general shape of a lesion.
2. The spectral principal component information of the color of the lesion area: transform the image from red-green-blue color space to P color space, and extract ten main color features of the images in P color space. Then the average pixels of each color feature in the three channels were calculated, and the median of all average pixels is the representative value of spectral principal component information. It was used to quantify the color characteristics of the lesion.
3. The image entropy of the S-channel in the HSI color space of the lesion area: transform the image from RGB color space to HSI color space and calculate the image entropy in the S-channel. It was another feature used for describing the color characteristics.
4. The texture information of the lesion area: the local binary patterns method was used to analyze the statistical texture features of an image. The change of texture information reflects the changes in the gastric mucosa.
5. The histogram of oriented gradients of the lesion area: the distribution (histograms) of directions of gradients (oriented gradients) are used as features. The edges and corners pack in a lot more information about object shape than flat regions. This index reflects information about the boundary and shape of a lesion.
6. The color moments of the lesion area: a simple but efficient color feature that reflects the general brightness, the distribution region of the color, and the symmetry of the color distribution.

**Fig. 3:**
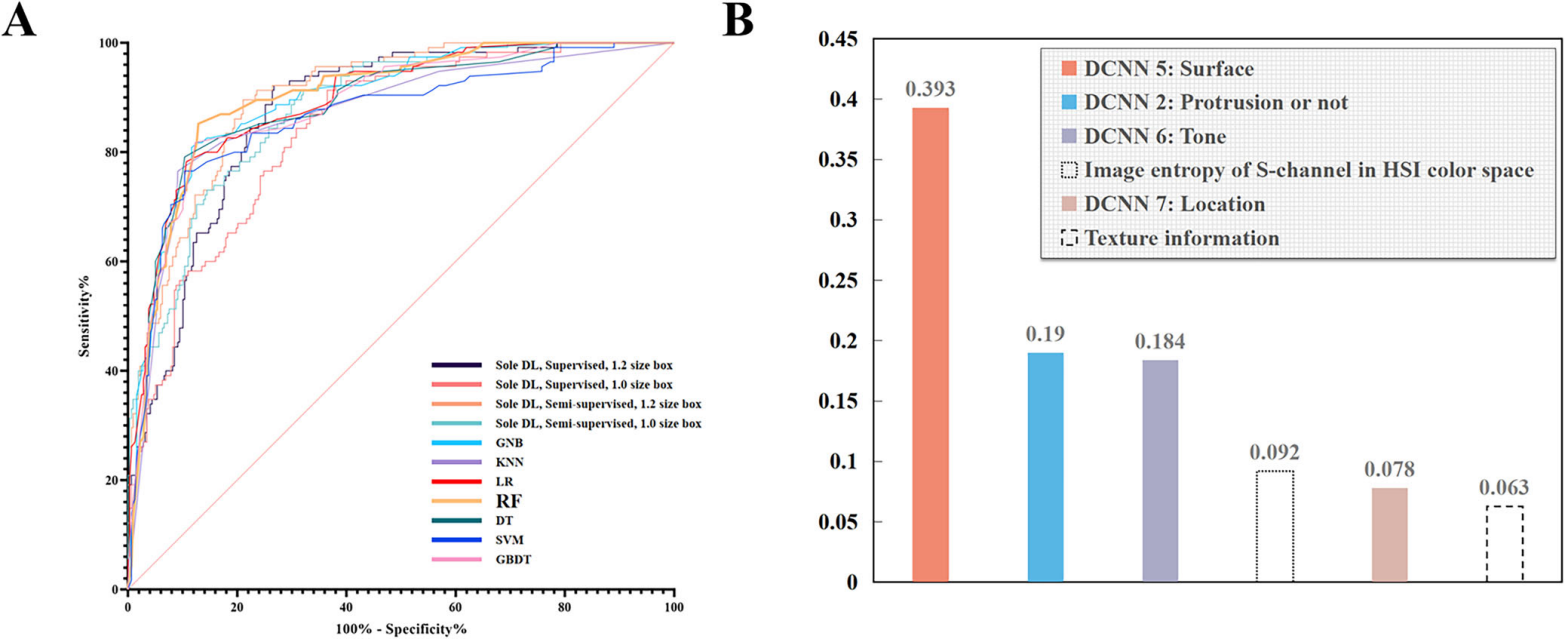
The performance of machine learning (ML) models and the weights of the included feature indexes. **A** The performance of the seven ML models on the internal image test set. Random forest (RF) showed the best performance. **B** Six indexes were determined by the RF model and the corresponding weights. RF, random forest, GNB, Gaussian Naive Bayes, KNN, k-Nearest Neighbor, LR, logistic regression, DT, decision tree, SVM, support vector machine, GBDT, gradient boosting decision tree.

When the seven DL-based features and six quantitative features were extracted, they were combined and inputted into the fitting diagnosis models using machine learning methods, including random forest (RF), Gaussian Naive Bayes (GNB), k-Nearest Neighbor (KNN), logistic regression (LR), decision tree (DT), support vector machine (SVM), and gradient boosting decision tree (GBDT). The best model was selected for constructing ENDOANGEL-ED. The representative images of these features and the schematic diagram of this study are shown in Fig. 3 and Supplementary Fig. 3. The literal workflow of this study is illustrated in Supplementary Fig. 4.

### Construction of sole DL model for diagnosing gastric neoplasms

The resnet-50 algorithm was used to construct a traditional sole DL model to diagnose early gastric neoplasms under WL using the same training set as ENDOANGEL-ED. We compared two image preprocessing methods (the detection box of the YOLO-V3 maintain the original size or be enlarged to 1.2 sized to contain more information about the mucosa around the lesion) and both supervised and semi-supervised algorithms in developing the sole DL model.

### Internal image test, external image test, internal video test, and external video test

The performance of the ENDOANGEL-ED and sole DL was tested in datasets 2–5 based on images and videos.

### Consecutive video test

The performance of ENDOANGEL-ED was tested in consecutive videos of patients undertaking EGD examinations from the RWHU between March 2022 and June 2022.

**Fig. 4:**
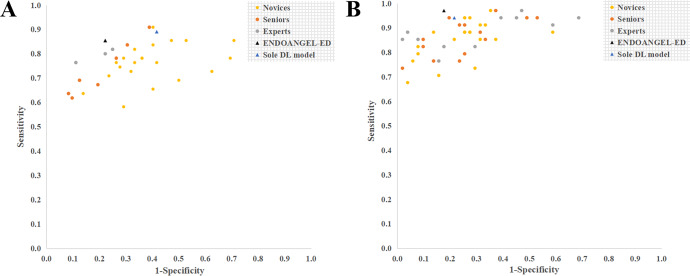
Performance of ENDOANGEL-ED and endoscopists in the internal and external videos. **A** Internal videos. **B** External videos.

The inclusion criteria were: (1) age ≥18 years; (2) sedated gastroscopy; and (3) can read, understand and sign informed consent. The exclusion criteria were: (1) emergency bleeding; (2) food residues; (3) history of gastrectomy or diagnosed as remnant stomach; and (4) no lesions or no pathology results. For enrolled patients, they were further selected according to the criteria for the lesions described above. Then the raw videos of the eligible lesions were collected. All the videos were edited into video clips containing target lesions. The ENDOANGEL-ED is activated when the image frame freeze. The prediction of the ultimately included features and the diagnosis by the ENDOANGEL-ED was presented on the screen (Fig. 4 and Video 1).

### Man–machine comparison

The man–machine comparison was conducted in the internal and external videos. Thirty-one endoscopists from the RWHU and 46 endoscopists from 44 other hospitals participated in the comparison in the internal and external videos, respectively. They independently reviewed all the video clips and answered “neoplastic” or “non-neoplastic.” The external man–machine comparison was re-analyzed from the trial we previously published^30^. The experience levels of the endoscopists were determined as novices [1–5 years of EGD (esophagogastroduodenoscopy) experience], seniors (6–10 years of EGD experience), and experts (>10 years of EGD experience). The performance of endoscopists was compared with ENDOANGEL-ED and sole DL.

### MRMC study

Thirty-one endoscopists and 127 video clips in the internal video test were involved in the MRMC study. Using a crossover design, we randomly and equally divided the endoscopists into group A (first read videos without ENDOANGEL-ED augmentation) and group B (first read videos with ENDOANGEL-ED augmentation). After a washout period of 2 weeks, the arrangement was reversed. The endoscopists had their own options to consider the augmentation or disregard it based on their judgment. The overall time of each endoscopist for reading these cases was recorded. The study design is shown in Supplementary Fig. 5.

### Acceptance analysis using a specific scale for the AI system

We modified and used a five-point Likert-type acceptance scale for the implementation of AI in gastrointestinal endoscopy published by Tian et al.^31^ The scale consisted of nine items for evaluating and comparing the trust, acceptance, confidence, etc., of endoscopists on the explainable AI system and the traditional sole DL system. Thirty-one endoscopists were invited to the scale evaluation. The scale form is attached in the Supplementary.

### Ethics

The Ethics Committee of RHWU approved this study. The institutional review boards exempted the informed consent for the retrospectively collected data. All the prospectively enrolled patients had signed the informed consent. The study was registered as ChiCTR2100045963 in the Chinese Clinical Trial Registry.

### Statistical analysis

As for the consecutive video test, the accuracy of ENDOANGEL-ED was estimated at 80%. The sample size was calculated as 72 with an alpha of 0.05 and a power of 0.80 using the Tests for One Proportion procedure (PASS 2021).

The performance of ENDOANGEL-ED, sole DL model, and endoscopists were evaluated by accuracy, sensitivity, specificity, PPV, and NPV. The McNemar test was used to compare the accuracy, sensitivity, and specificity. The χ^2^ test was used to compare the PPV and NPV between ENDOANGEL-ED and the sole DL model. The inter-rater agreement among the endoscopists was calculated using Fleiss’ Kappa. Performance metrics between different levels of endoscopists and ENDOANGEL-ED and the sole DL model were compared using the Mann–Whitney U test. The comparison of the acceptance and other items in the questionnaire was analyzed using Wilcoxon signed-rank Test. *P* values <0.05 were considered statistically significant.

### Reporting summary

Further information on research design is available in the Nature Research Reporting Summary linked to this article.

## Supplementary information

Video 1

Supplementary file

REPORTING SUMMARY

## Data Availability

Individual de-identified participant data and pretraining model, software, and source code reported in this article will be shared with investigators after article publication. Data requesters could contact the corresponding author H.G.Y. (yuhonggang@whu.edu.cn) to gain access.

## Code availability

The code may be made available upon reasonable request to L.L.W. (wu_leanne@163.com).
