# Publisher Correction: Explainable artificial intelligence incorporated with domain knowledge diagnosing early gastric neoplasms under white light endoscopy

**DOI:** 10.1038/s41746-023-00855-2

**Published:** 2023-06-06

**Authors:** Zehua Dong, Junxiao Wang, Yanxia Li, Yunchao Deng, Wei Zhou, Xiaoquan Zeng, Dexin Gong, Jun Liu, Jie Pan, Renduo Shang, Youming Xu, Ming Xu, Lihui Zhang, Mengjiao Zhang, Xiao Tao, Yijie Zhu, Hongliu Du, Zihua Lu, Liwen Yao, Lianlian Wu, Honggang Yu

**Affiliations:** 1 Renmin Hospital of Wuhan University, Wuhan, China; 2 Key Laboratory of Hubei Province for Digestive System Disease, Renmin Hospital of Wuhan University, Wuhan, China; 3 Hubei Provincial Clinical Research Center for Digestive Disease Minimally Invasive Incision, Renmin Hospital of Wuhan University, Wuhan, China; 4 Department of Gastroenterology, Wenzhou Central Hospital, Wenzhou, China; 5 Department of Gastroenterology, The Central Hospital of Wuhan, Tongji Medical College, Huazhong University of Science and Technology, Wuhan, China

**Keywords:** Gastric cancer, Experimental models of disease

Correction to: *npj Digital Medicine* 10.1038/s41746-023-00813-y, published online 12 April 2023

In this article the legend for Figure legends for 1 to 4 were incorrectly matched. The figure legends should have appeared as shown below. The original article has been corrected.

**Fig. 1:**
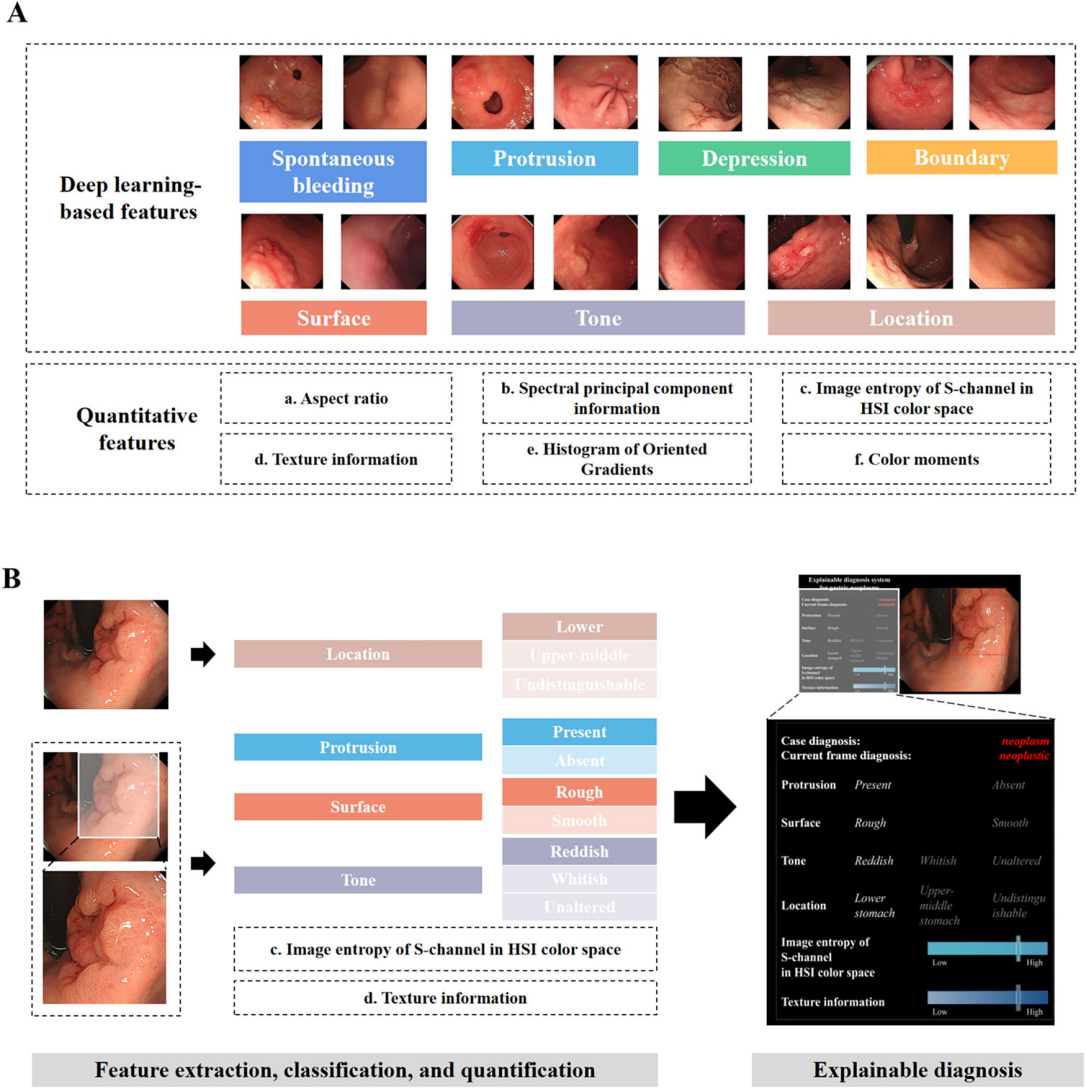
The schematic diagram of all feature indexes and the framework of developing ENDOANGEL-ED. **A** Thirteen features, including seven deep learning-based features and six quantitative features. **B** The framework of developing ENDOANGEL-ED. HIS Hue, Saturation, Intensity.

**Fig. 2:**
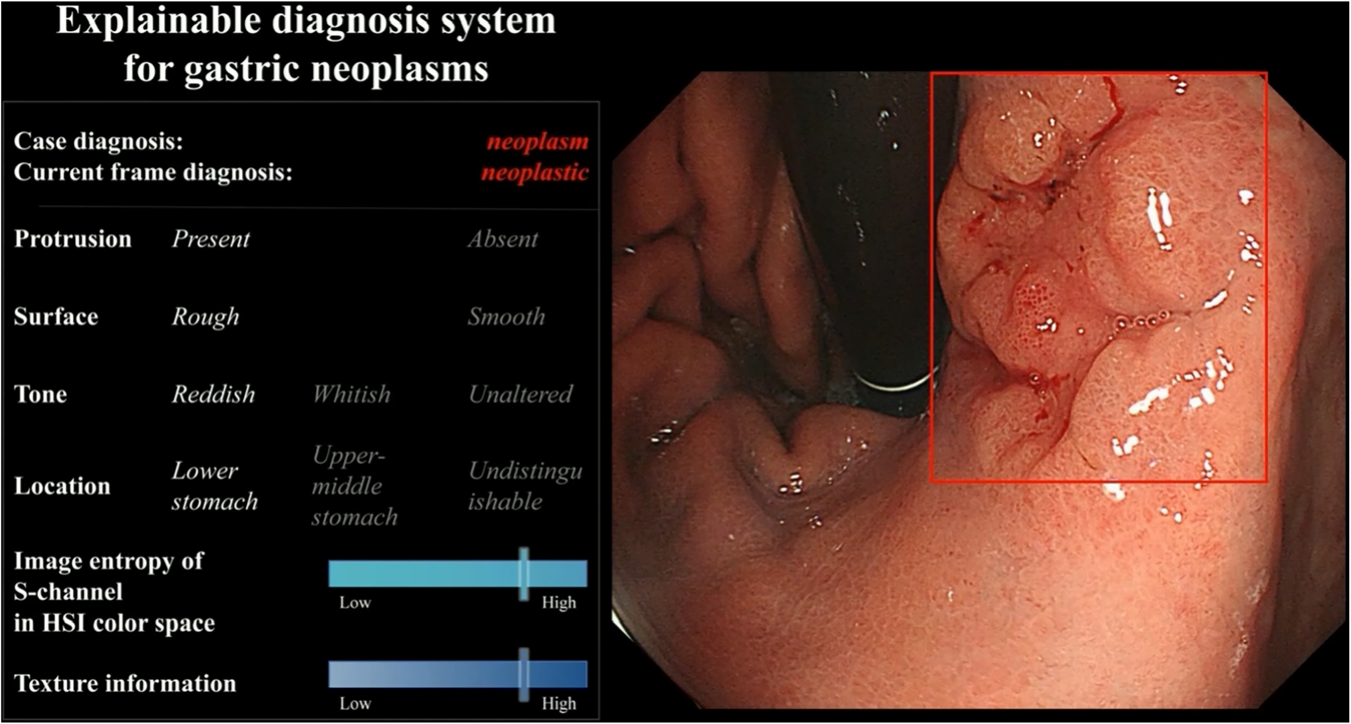
The system interface of ENDOANGEL-ED. The prediction of the six feature indexes and the diagnostic result were presented on the left.

**Fig. 3:**
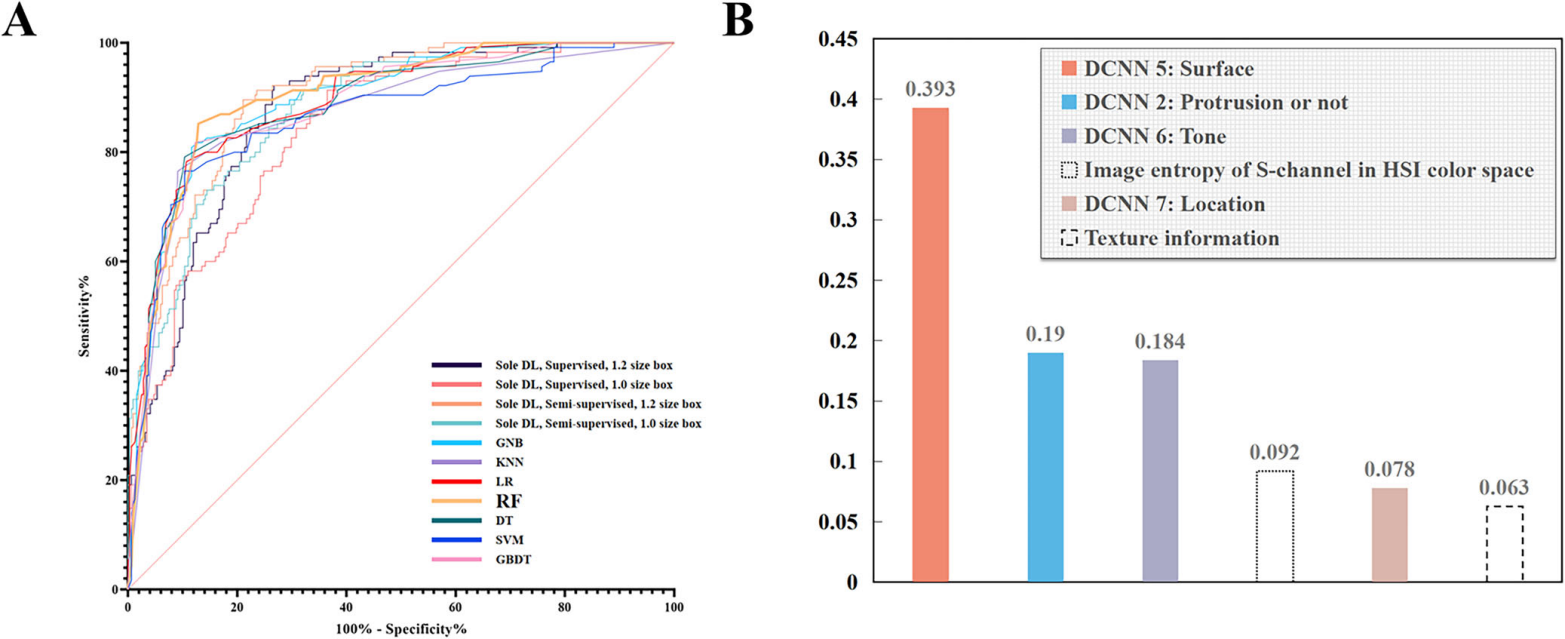
The performance of machine learning (ML) models and the weights of the included feature indexes. **A** The performance of the seven ML models on the internal image test set. Random forest (RF) showed the best performance. **B** Six indexes were determined by the RF model and the corresponding weights. RF random forest, GNB Gaussian Naive Bayes, KNN k-Nearest Neighbor, LR logistic regression, DT decision tree, SVM support vector machine, GBDT gradient boosting decision tree.

**Fig. 4:**
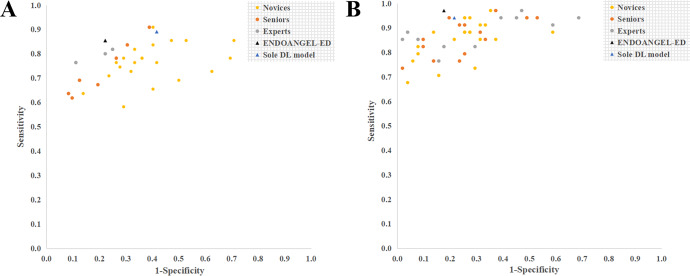
Performance of ENDOANGEL-ED and endoscopists in the internal and external videos. **A** Internal videos. **B** External videos.

