# Supplementary material for: Explainable artificial intelligence incorporated with domain knowledge diagnosing early gastric neoplasms under white light endoscopy
Source: NPJ Digit Med. 2023 Apr 12;6:64. doi: 10.1038/s41746-023-00813-y (PMC10097818; doi:10.1038/s41746-023-00813-y)
Supplement: Supplementary file 2 — Supplementary file [file 41746_2023_813_MOESM2_ESM.pdf]

Explainable artificial intelligence incorporated with domain knowledge to diagnose early gastric neoplasms under white light endoscopy: A multi-center study

The training, validation, and testing of DCNN 1-7

DCNN 1-6 were newly constructed models in the present work applied both semi-supervised and supervised algorithms. As for the semi-supervised models, the basic framework of the Mean Teacher algorithm was shown in Figure S11. All the six DCNN were trained, validated, and tested using images in dataset 1 (3,612 white light images containing focal lesions).

DCNN 1: For determining whether a lesion has spontaneous bleeding, DCNN 1 was trained by 150 images with spontaneous bleeding and 720 images without spontaneous bleeding. In the test set, 38 images with spontaneous bleeding and 183 images without spontaneous bleeding were used. ResNet-50[1] achieved an accuracy of 83.26% (77.78%-87.60%) by Supervised algorithms while achieved an accuracy of 94.57% (90.75%-96.87%) by Semi-supervised algorithms.

DCNN 2: For distinguishing whether a lesion is protuberant or not, DCNN 2 was trained by 706 protuberant images and 732 non-protuberant images. In the test set, 185 protuberant images and 186 non-protuberant images were used. ResNet-50 achieved an accuracy of 81.40% (77.13%-85.03%) by Supervised algorithms while achieved an accuracy of 85.44% (81.49%-88.67%) by Semi-supervised algorithms.

DCNN 3: For distinguishing whether a lesion is depressed or not, DCNN 3 was trained by 1230 depressed images and 1410 non-depressed images. In the test set, 306 depressed images and 352 non-depressed images were used. ResNet-50 achieved an accuracy of 74.32% (70.85%-77.51%) by Supervised algorithms while achieved an accuracy of 76.90% (73.53%-79.96%) by Semi-supervised algorithms.

DCNN 4: For determining whether a lesion has a clear boundary, DCNN 4 was trained by 2096 images with clear boundary and 789 images without clear boundary. In the test set, 529 images with clear boundary and 198 images without clear

boundary were used. ResNet-50 achieved an accuracy of 72.63% (69.28%-75.75%) by Supervised algorithms while achieved an accuracy of 75.52% (72.27%-78.51%) by Semi-supervised algorithms.

DCNN 5: For distinguishing whether the surface of a lesion is rough or smooth, DCNN 5 was trained by 401 images with rough surface lesion and 2206 images with smooth surface lesion. In the test set, 104 images with rough surface lesion and 556 images with smooth surface lesion were used. ResNet-50[1] achieved an accuracy of 78.79% (75.51%-81.74%) by Supervised algorithms while achieved an accuracy of 81.97% (78.85%-84.72%) by Semi-supervised algorithms.

DCNN 6: For distinguishing whether the tone of a lesion is reddish, whitish, or the same as the background mucosa, DCNN 6 was trained by 1243 images with reddish tone of the lesion and 205 images with whitish tone of the lesion and 791 images with unaltered tone as the background mucosa. In the test set, 321 images with reddish tone of the lesion and 45 images with whitish tone of the lesion and 212 images with unaltered tone were used. ResNet-50 achieved an accuracy of 70.93% (67.10%-74.48%) by Supervised algorithms while achieved an accuracy of 81.31% (77.93%-84.28%) by Semi-supervised algorithms.

Two models were constructed in our previously related works. The YOLO-v3[2] was trained to locate the lesion in real-time. 21,000 gastric images were selected and labeled by endoscopists for training and validation: 15,341 images with lesions and 5659 normal control images. YOLO-v3 showed a sensitivity of 96.9% and 95.6% in 2,733 and 8,450 internal and external still images, and showed a sensitivity of 92.8% in 3,684 lesions of 1,774 prospective patients.[3] DCNN 7 was trained to identify 26 anatomical landmarks of EGC examination. 75742 images (with more than 2000 images in each landmark) were used for training and validation. 7290 images (with 270 images on each landmark) were used for testing. ResNet-50 achieved an overall accuracy of 93.8% in the test set. The accuracy of this model in internal, external, and prospective videos were 95.3%, 95.3, and 95.2%, respectively.[4]

Scale form

**I.** Using AI will reduce your workload.					
Explainable AI	☐Strongly disagree	☐Disagree	☐Neutral	☐Agree	☐Strongly agree
Traditional DL model	☐Strongly disagree	☐Disagree	☐Neutral	☐Agree	☐Strongly agree
**II.** The use of AI will not affect you for focusing on improving the ability of diagnosis.					
Explainable AI	☐Strongly disagree	☐Disagree	☐Neutral	☐Agree	☐Strongly agree
Traditional DL model	☐Strongly disagree	☐Disagree	☐Neutral	☐Agree	☐Strongly agree
**III.** Using AI will make you more interested in lesion observation and diagnosis.					
Explainable AI	☐Strongly disagree	☐Disagree	☐Neutral	☐Agree	☐Strongly agree
Traditional DL model	☐Strongly disagree	☐Disagree	☐Neutral	☐Agree	☐Strongly agree
**IV.** Using AI will allow you to focus more on lesion observation and diagnosis.					
Explainable AI	☐Strongly disagree	☐Disagree	☐Neutral	☐Agree	☐Strongly agree
Traditional DL model	☐Strongly disagree	☐Disagree	☐Neutral	☐Agree	☐Strongly agree
**V.** Using AI will increase the patients' trust in your diagnosis.					
Explainable AI	☐Strongly disagree	☐Disagree	☐Neutral	☐Agree	☐Strongly agree
Traditional DL model	☐Strongly disagree	☐Disagree	☐Neutral	☐Agree	☐Strongly agree
**VI.** Your level of trust in using AI.					
Explainable AI	☐Very low	☐Low	☐Neutral	☐High	☐Very High
Traditional DL model	☐Very low	☐Low	☐Neutral	☐High	☐Very High
**VII.** Your acceptance of AI.					
Explainable AI	☐Very low	☐Low	☐Neutral	☐High	☐Very High
Traditional DL	☐Very low	☐Low	☐Neutral	☐High	☐Very High

model					
**VIII.** Using AI will bring you psychological comfort.					
Explainable AI	☐Strongly disagree	☐Disagree	☐Neutral	☐Agree	☐Strongly agree
Traditional DL model	☐Strongly disagree	☐Disagree	☐Neutral	☐Agree	☐Strongly agree
**IX.** AI systems will remind you to think more comprehensively.					
Explainable AI	☐Strongly disagree	☐Disagree	☐Neutral	☐Agree	☐Strongly agree
Traditional DL model	☐Strongly disagree	☐Disagree	☐Neutral	☐Agree	☐Strongly agree

Reference

1. Akiba T, Suzuki S, Fukuda KJapa. Extremely large minibatch sgd: Training resnet-50 on imagenet in 15 minutes. arXiv preprint arXiv. 2017.
2. Redmon J, Divvala S, Girshick R, Farhadi A. You only look once: Unified, real-time object detection. Proceedings of the IEEE conference on computer vision and pattern recognition.2016.
3. Wu L, Xu M, Jiang X, et al. Real-time artificial intelligence for detecting focal lesions and diagnosing neoplasms of the stomach by white-light endoscopy (with videos). *Gastrointest Endosc* 2022; **95**(2): 269-80.e6.
4. Dong Z, Wu L, Mu G, et al. A deep learning-based system for real-time image reporting during esophagogastroduodenoscopy: a multicenter study. *Endoscopy* 2022; **54**(8): 771-7.

Supplementary Table 1. The test performance of feature-extraction models (DCNN 1-6)

Models	Function	Accuracy of Supervised algorithms (95% CI)	Accuracy of Semi-supervised algorithms (95% CI)
DCNN 1	Determining whether a lesion has spontaneous bleeding	83.26% (77.78%-87.60%)	94.57% (90.75%-96.87%) ***
DCNN 2	Distinguishing whether a lesion is protuberant or not	81.40% (77.13%-85.03%)	85.44% (81.49%-88.67%)
DCNN 3	Distinguishing whether a lesion is depressed or not	74.32% (70.85%-77.51%)	76.90% (73.53%-79.96%)
DCNN 4	Determining whether a lesion has a clear boundary	72.63% (69.28%-75.75%)	75.52% (72.27%-78.51%)
DCNN 5	Distinguishing whether the surface of a lesion is rough or smooth	78.79% (75.51%-81.74%)	81.97% (78.85%-84.72%)
DCNN 6	Distinguishing whether the tone of a lesion is red, pale, or the same as the background mucosa	70.93% (67.10%-74.48%)	81.31% (77.93%-84.28%) ***

Table legend

Supplementary Table 1. The test performance of feature-extraction models (DCNN 1-6). *Significant difference between the supervised and semi-supervised algorithm ($p < 0.05$) . The McNemar test was used to compare the accuracy of supervised and semi-supervised algorithms.

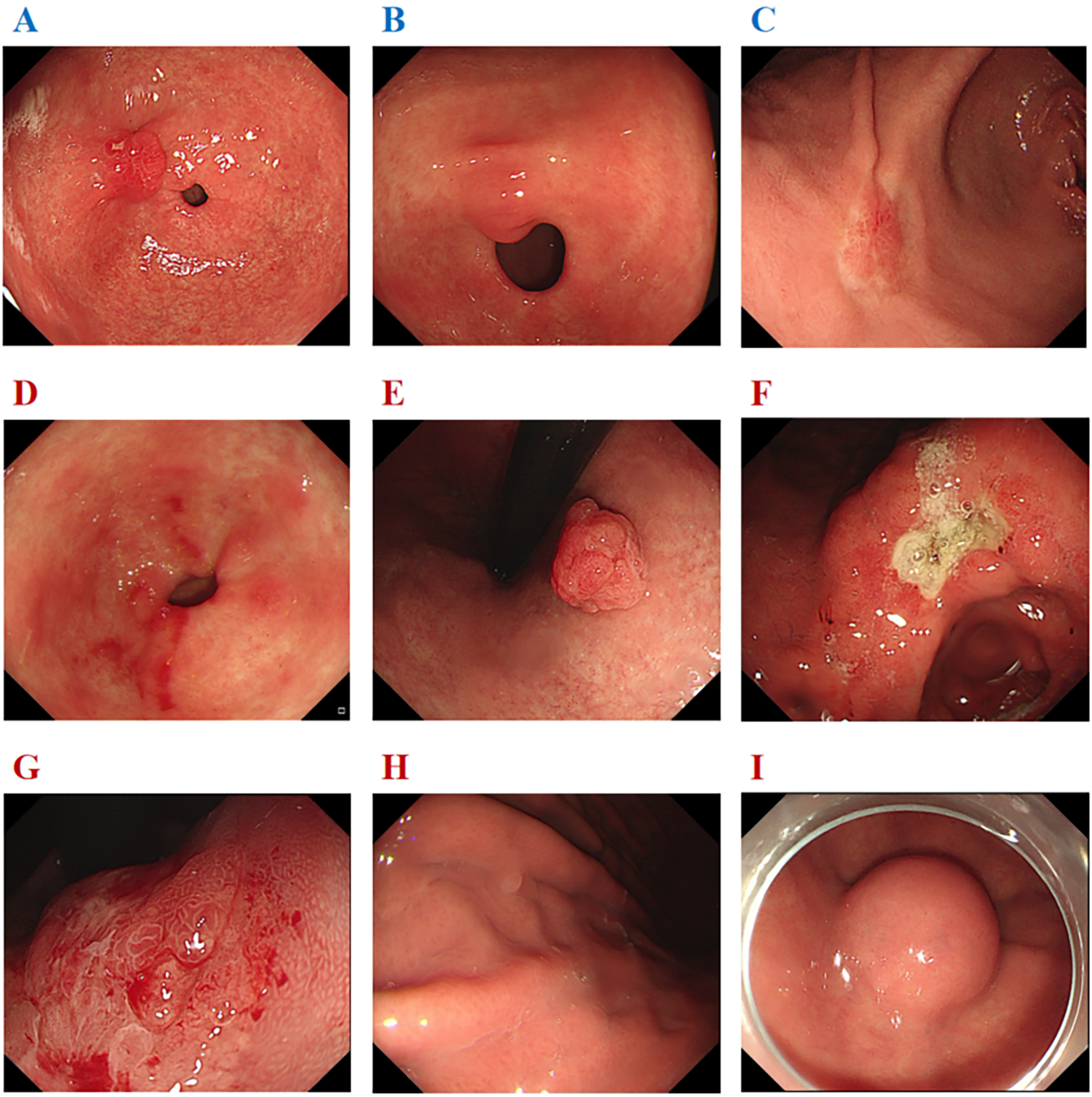

Supplementary Figure 1. Representative eligible and ineligible images of lesions. A, B, and C were eligible focal lesions. D, E, and F were ineligible due to multiple lesions, type I and type III lesions. G, H, and I were ineligible due to the field of view being too close or too far and submucosal lesions.

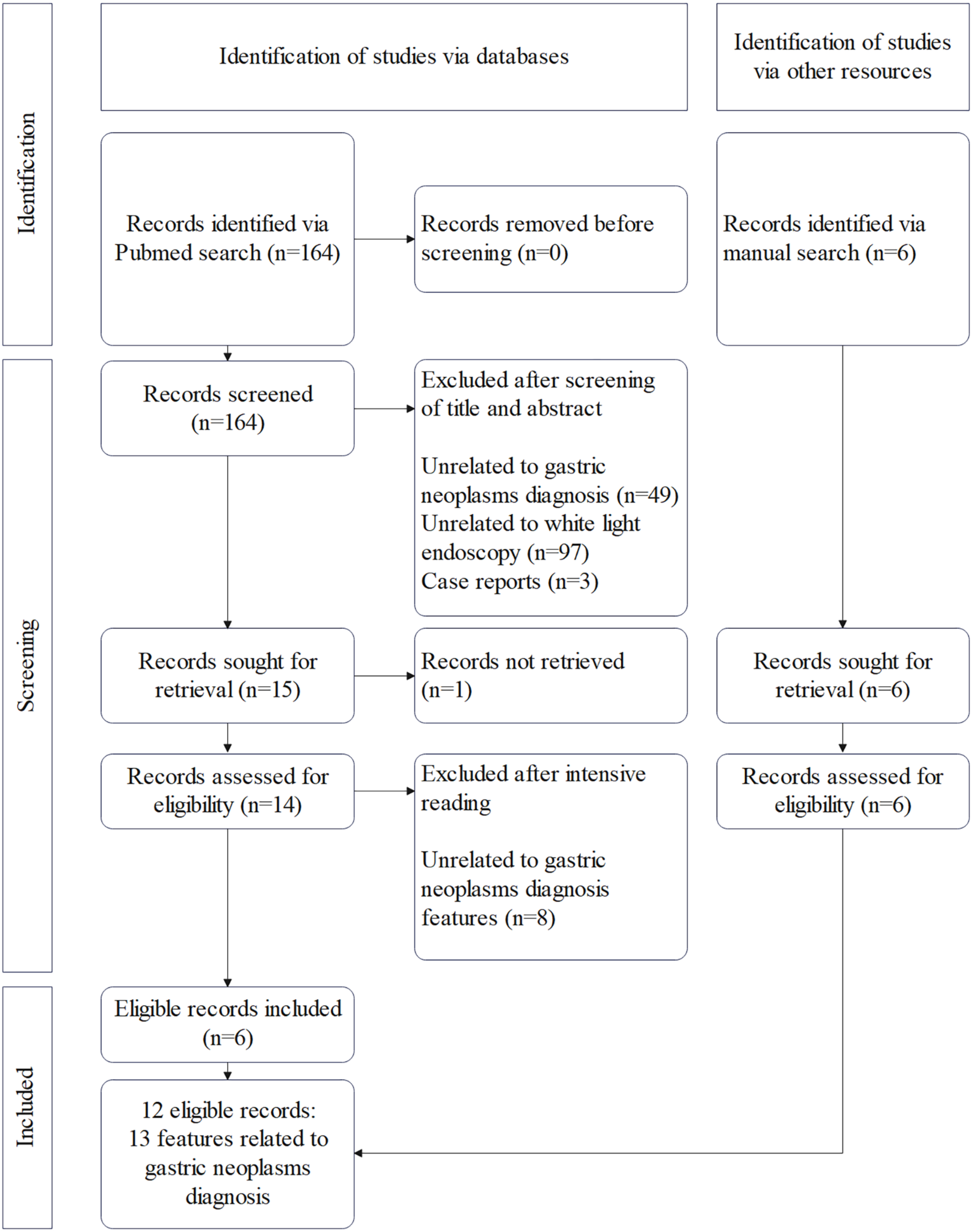

Supplementary Figure 2. The flow diagram of the literature research for the feature indexes related to gastric neoplasms at the early stage.

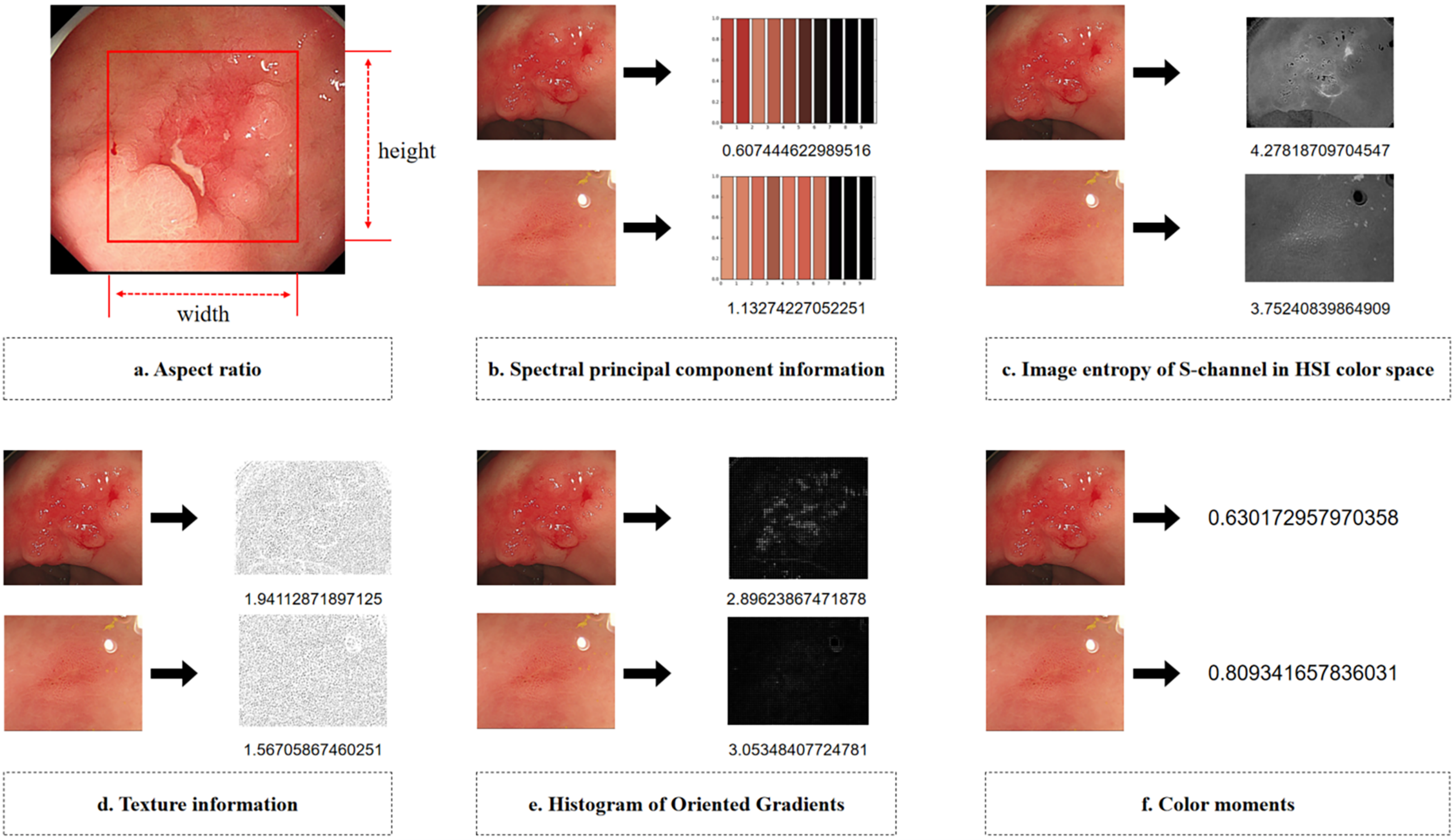

Supplementary Figure 3. The representative images for the six quantitative features.

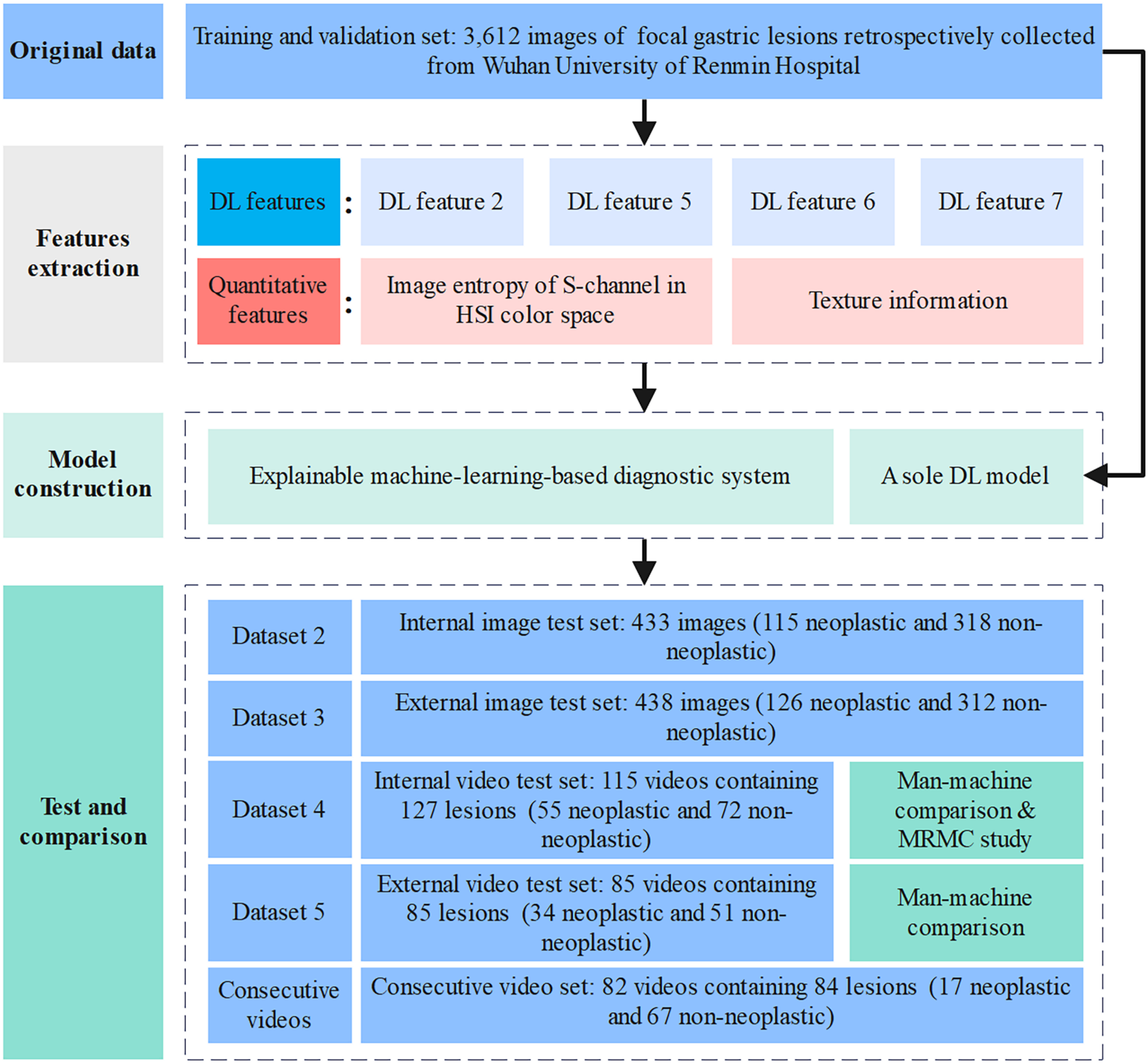

Supplementary Figure 4. The literal workflow of this study. DL, deep learning. MRMC, multi-reader multi-case.

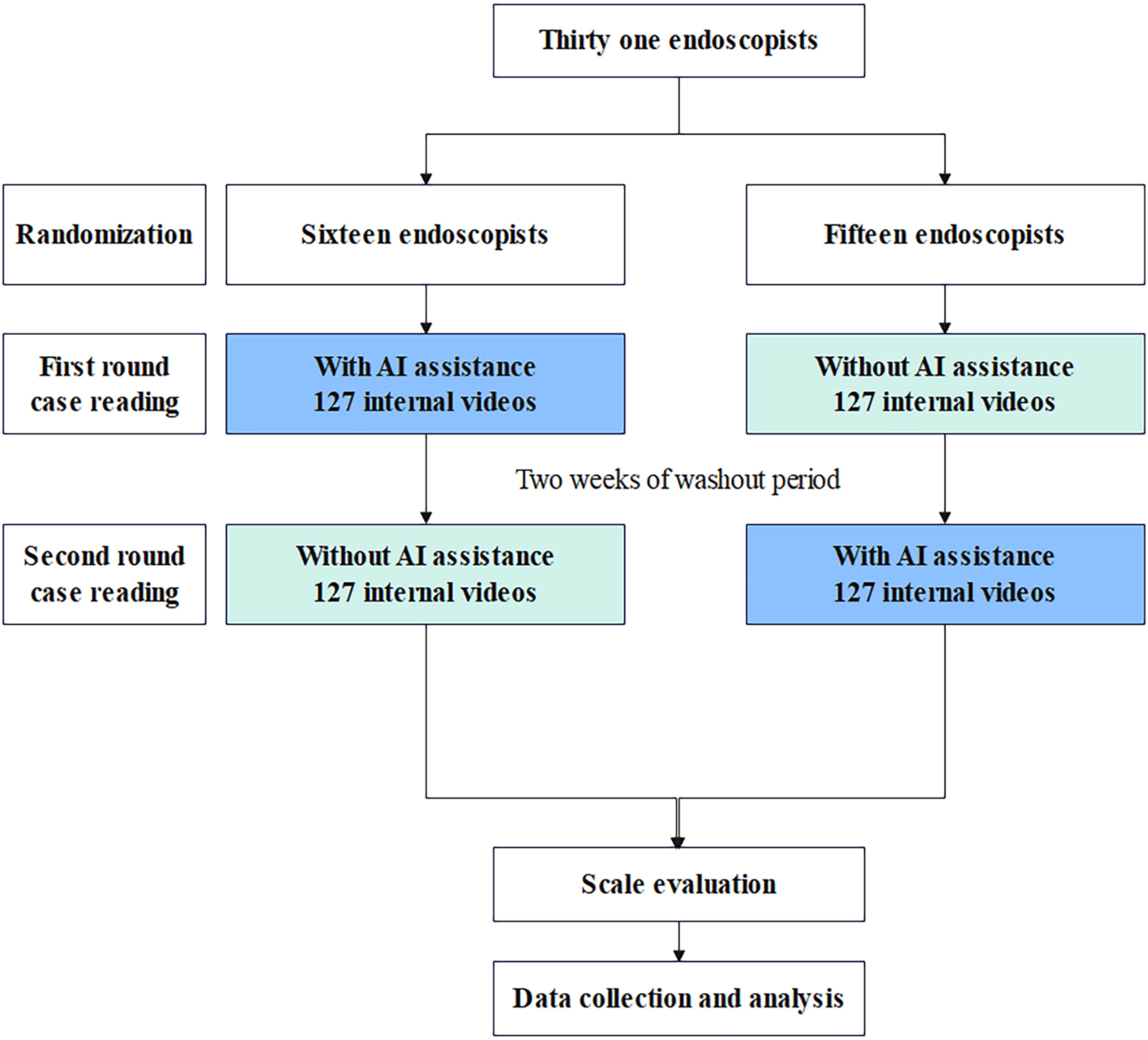

Supplementary Figure 5. The flow diagram of the multi-reader multi-case study.

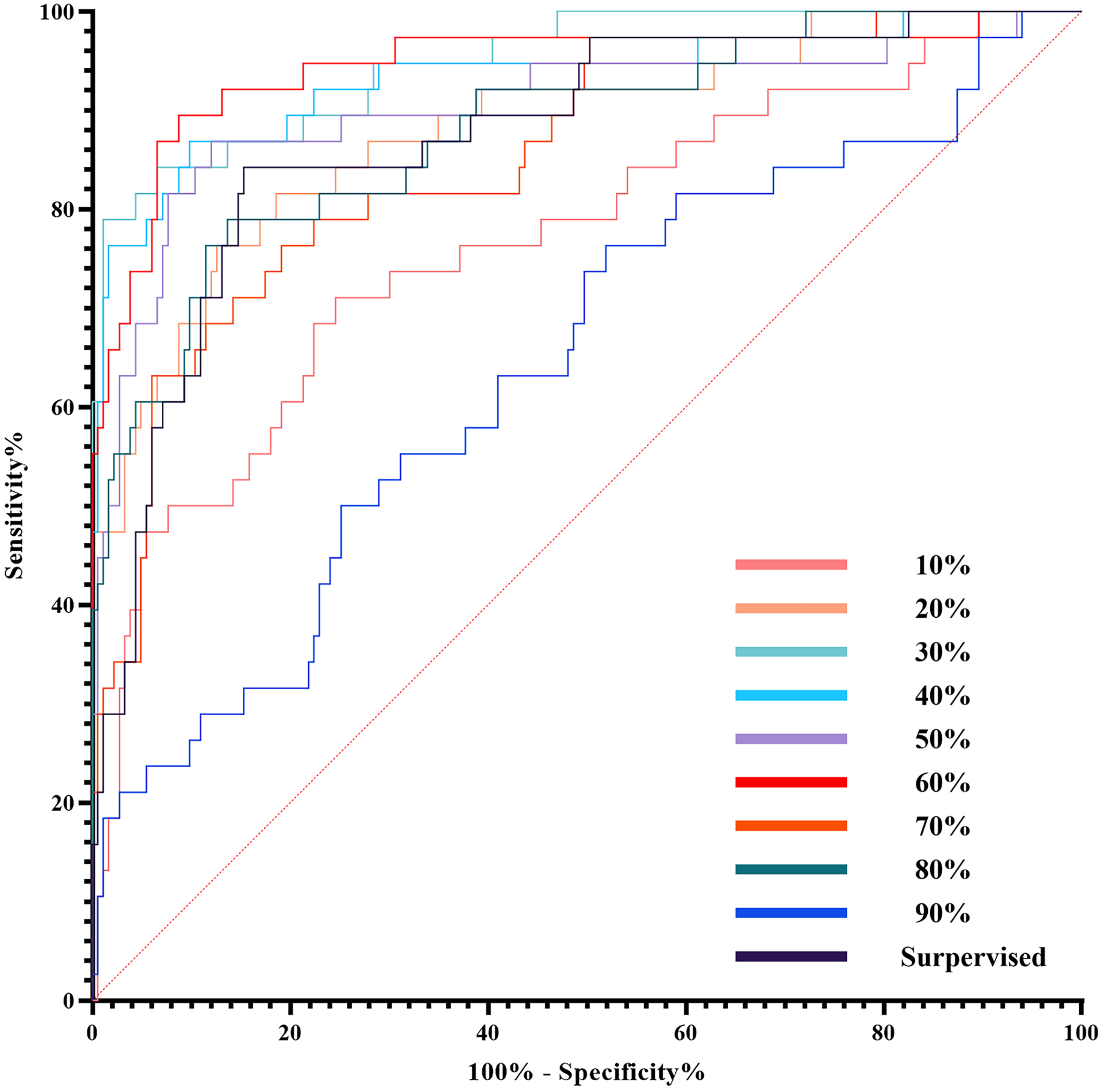

Supplementary Figure 6. Performance of the semi-supervised and supervised models for distinguishing spontaneous bleeding or not. As for the semi-supervised models, 10% to 90% (increase by 10%) of the original training set was used for training. Nine semi-supervised models and one supervised model using the original training set for model development were then tested on the same test set.

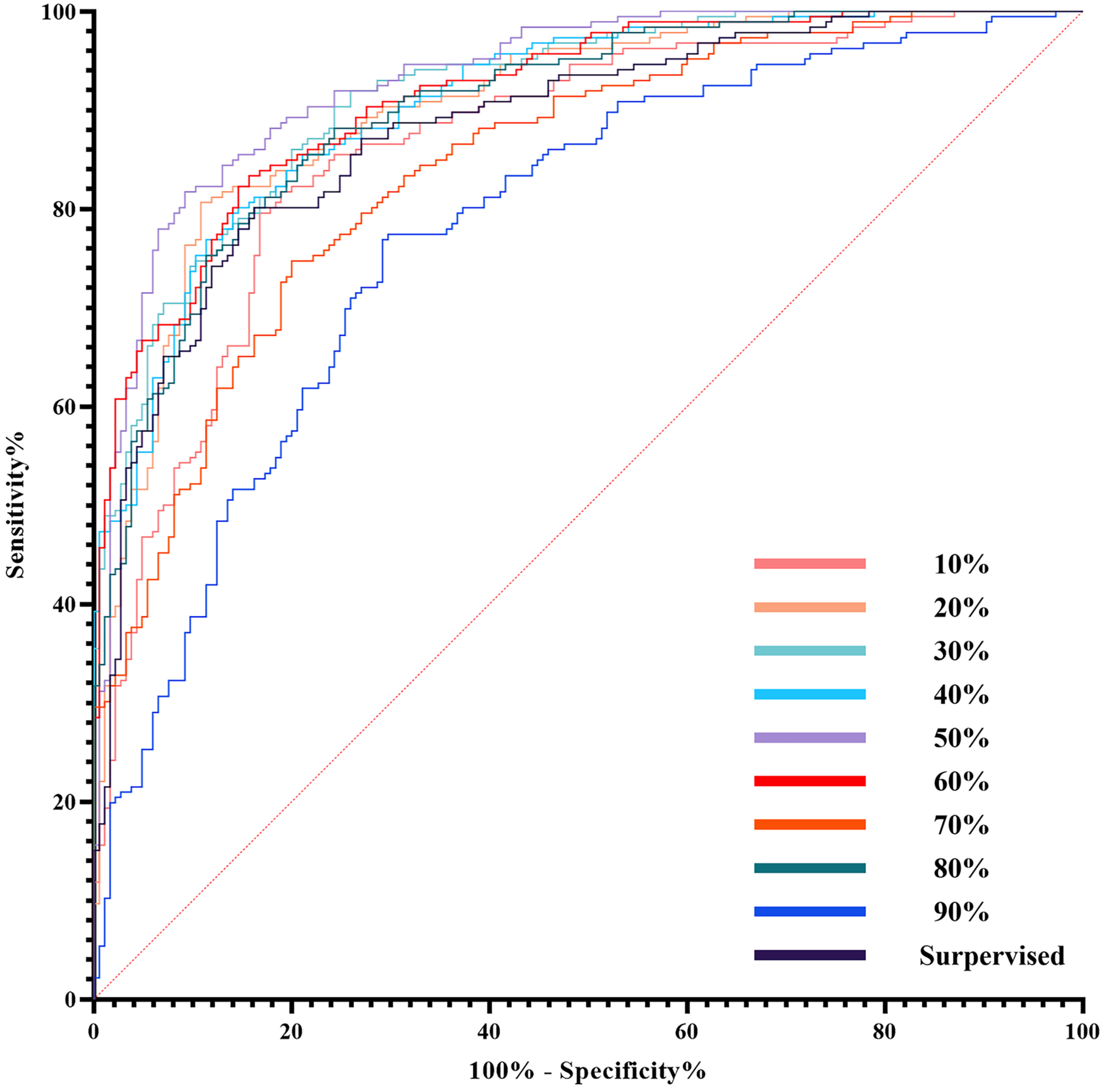

Supplementary Figure 7. Performance of the semi-supervised models and supervised model for distinguishing protrusion or not. As for the semi-supervised models, 10% to 90% (increase by 10%) of the original training set was used for training. Nine semi-supervised models and one supervised model using the original training set for model development were then tested on the same test set.

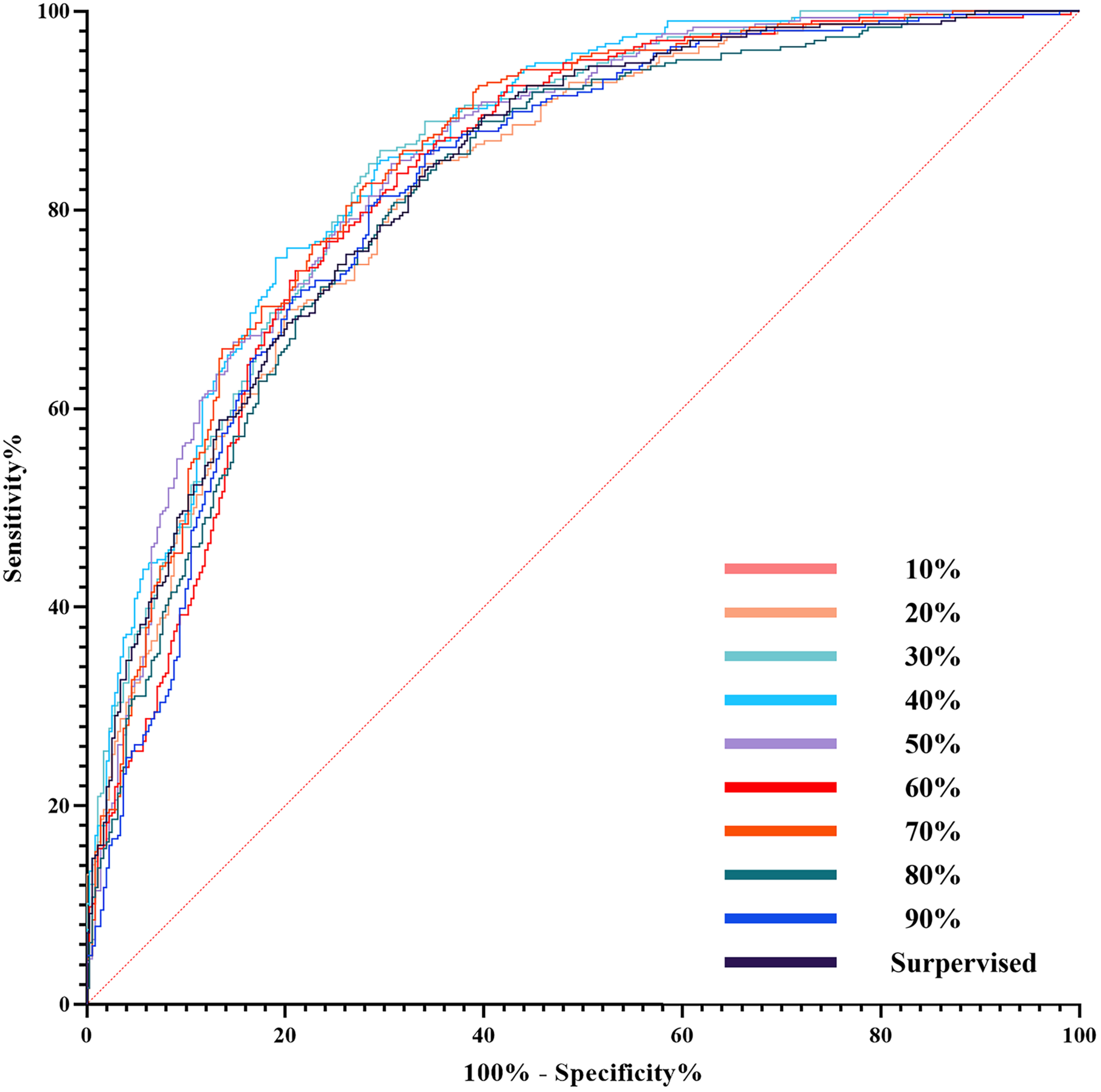

Supplementary Figure 8. Performance of the semi-supervised models and supervised model for distinguishing depression or not. As for the semi-supervised models, 10% to 90% (increase by 10%) of the original training set was used for training. Nine semi-supervised models and one supervised model using the original training set for model development were then tested on the same test set.

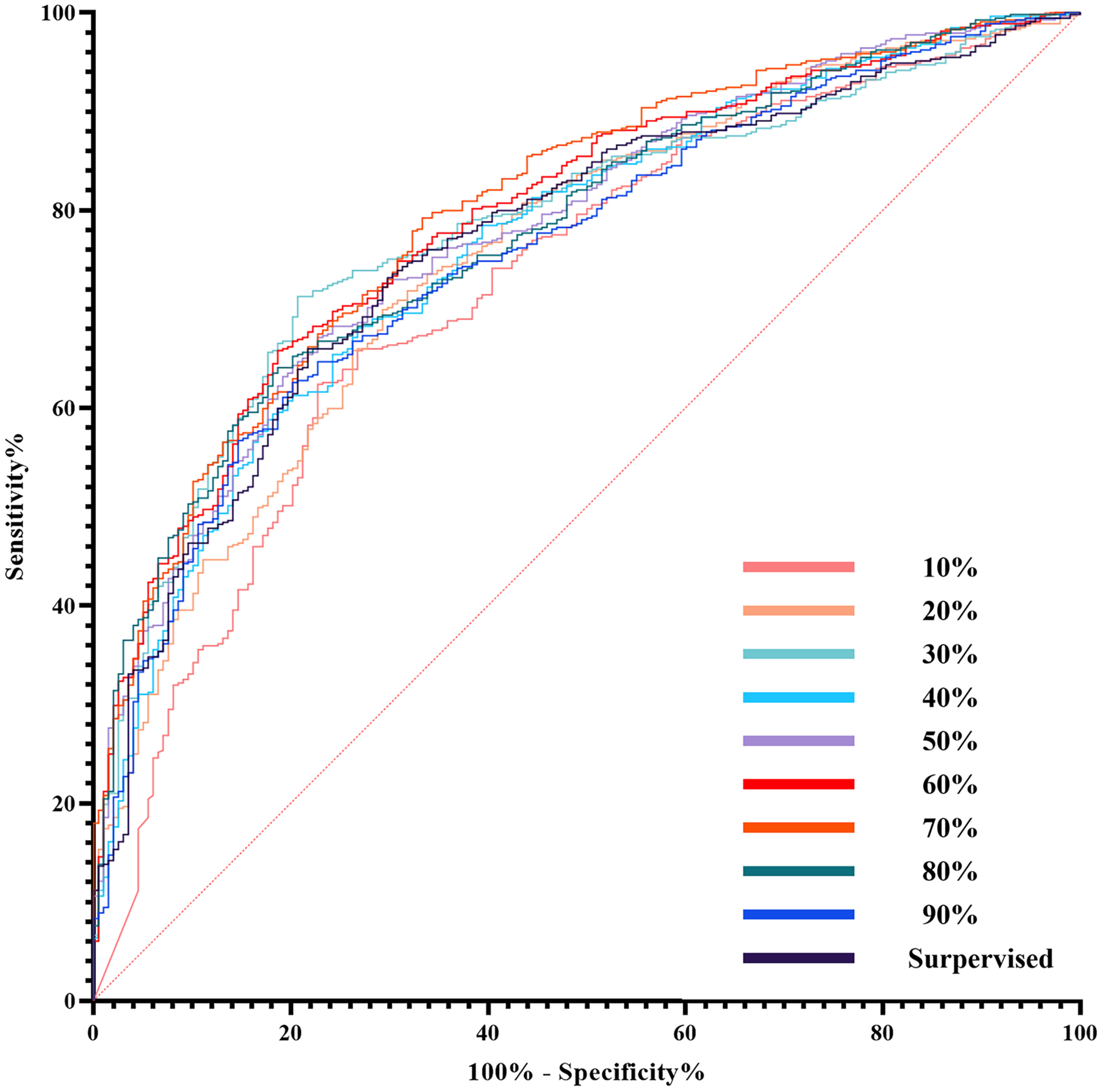

Supplementary Figure 9. Performance of the semi-supervised models and supervised models for identifying whether the boundary of a lesion is present or not. As for the semi-supervised models, 10% to 90% (increase by 10%) of the original training set was used for training. Nine semi-supervised models and one supervised model using the original training set for model development were then tested on the same test set.

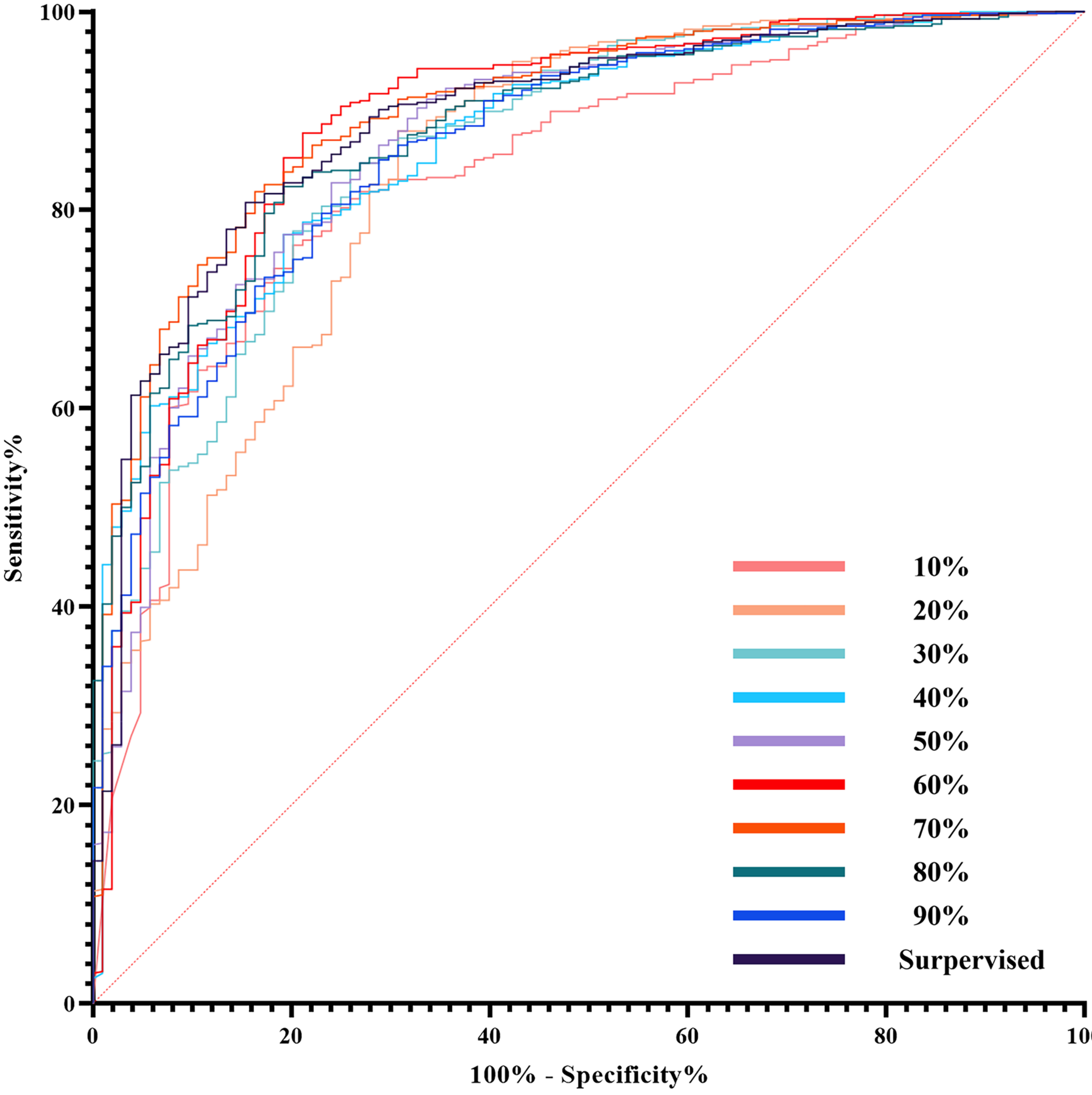

Supplementary Figure 10. Performance of the semi-supervised and supervised models for identifying whether the surface of a lesion is rough or smooth. As for the semi-supervised models, 10% to 90% (increase by 10%) of the original training set was used for training. Nine semi-supervised models and one supervised model using the original training set for model development were then tested on the same test set.

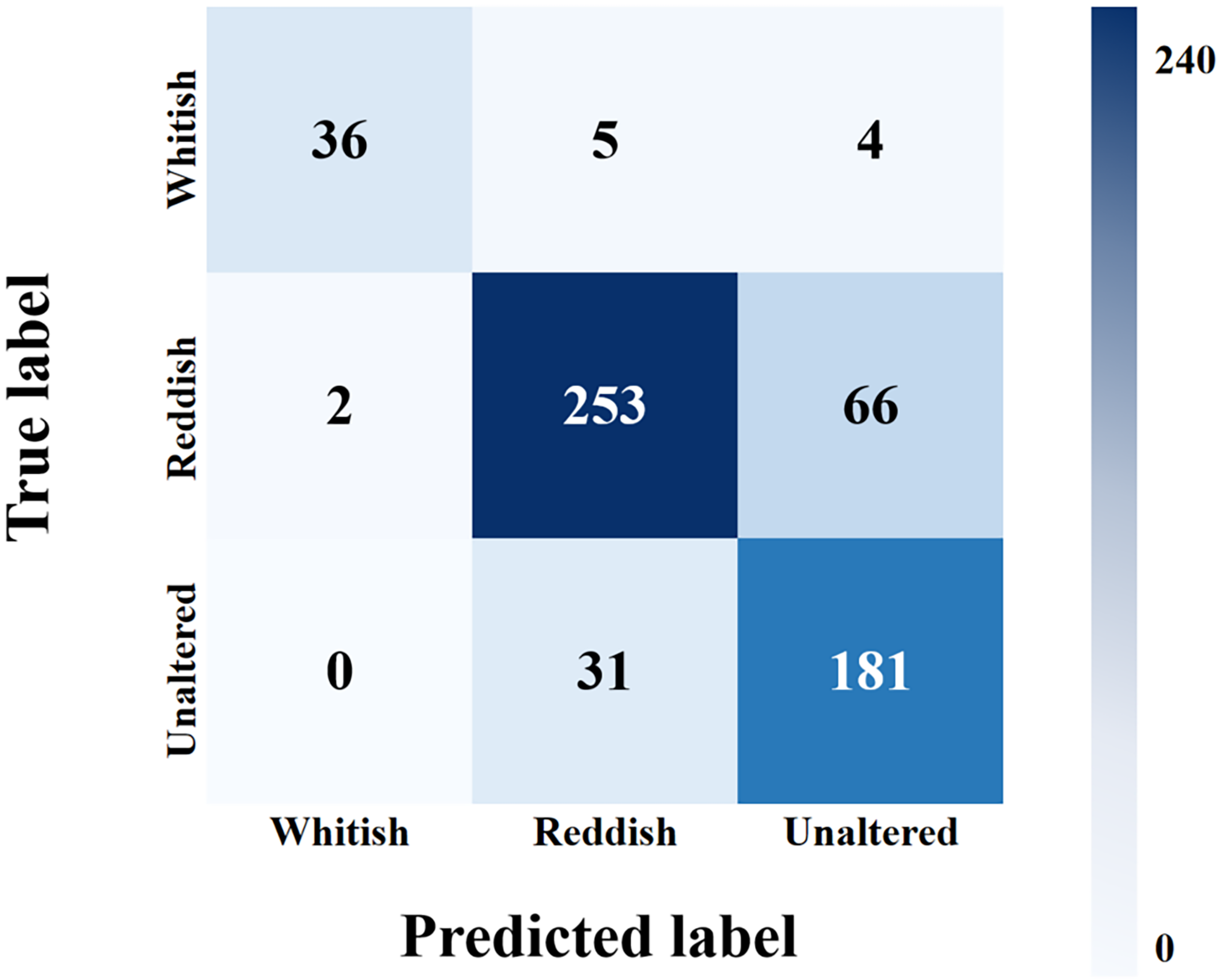

Supplementary Figure 11. Performance of the best semi-supervised model for determining the tone of the lesion.

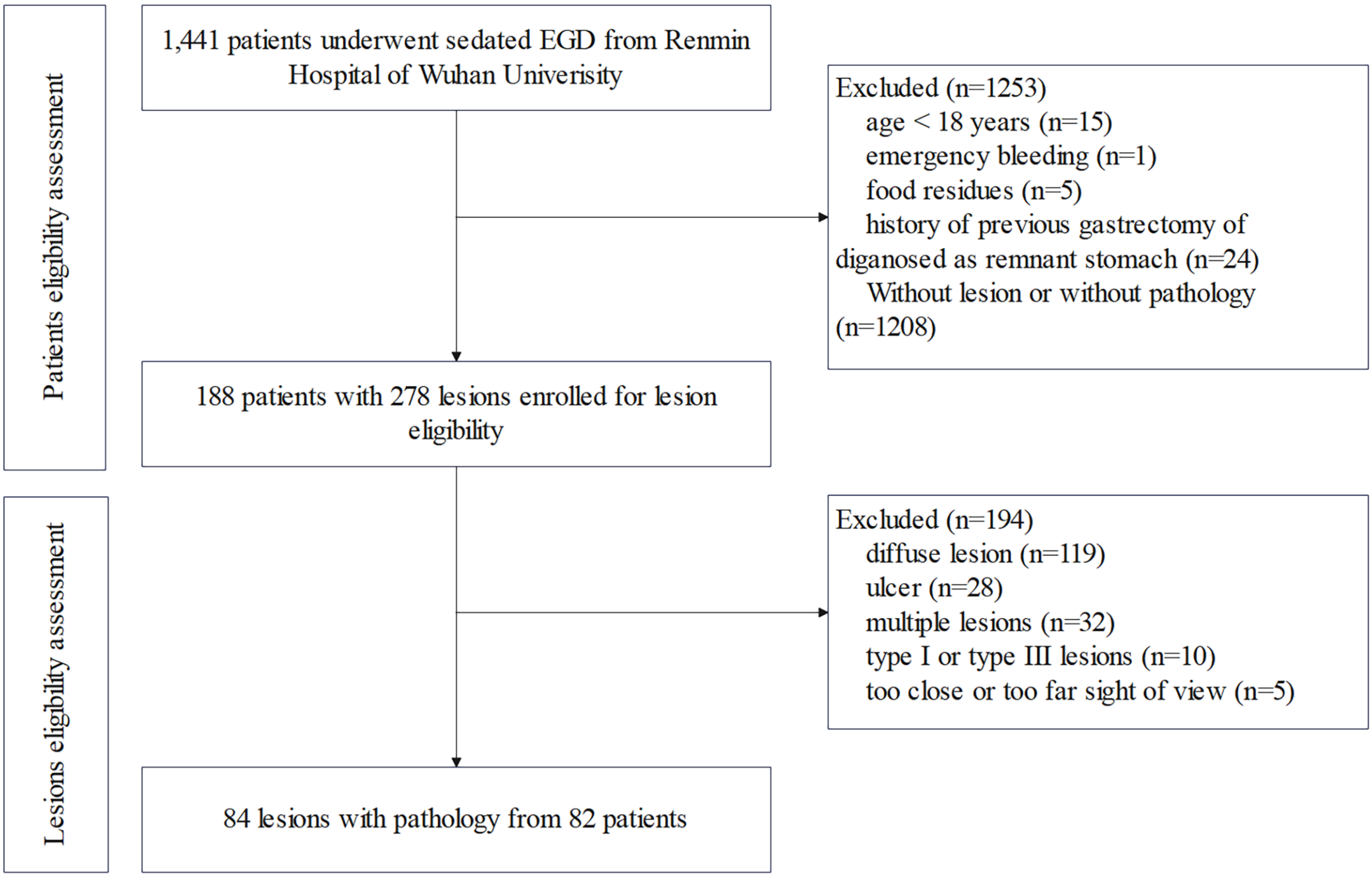

Supplementary Figure 12. The flow diagram of the eligibility of the patients and lesions in the consecutive video test.

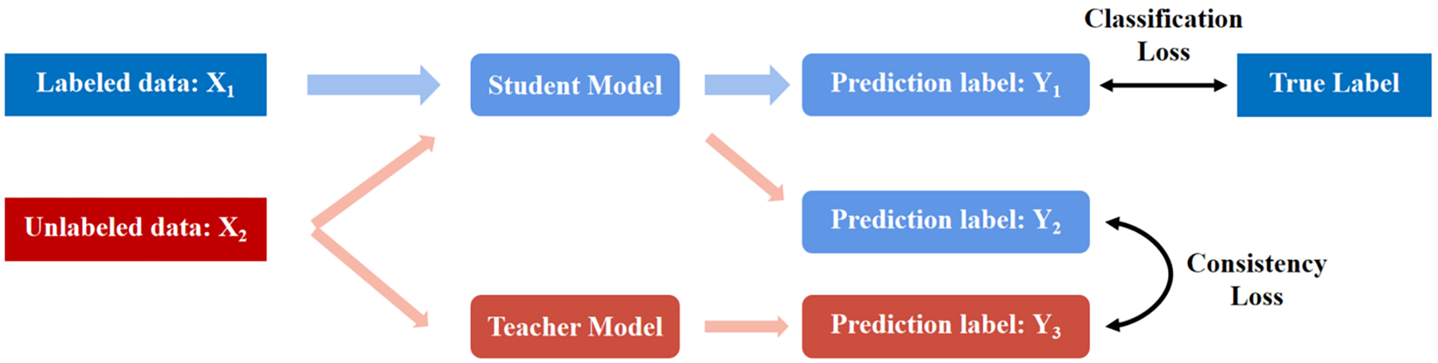

Supplementary Figure 13. The framework of the Mean Teacher algorithm for the construction of semi-supervised models. The Mean Teacher method was used for the construction of the semi-supervised models. The teacher model is initialized with the student model. The figure depicts a training batch with labeled data X1. Specifically, the softmax output Y1 of the student model is compared with the true label using classification loss. Besides, the unlabeled data X2 is fed in both the student and teacher models, obtaining the prediction results Y2 and Y3, respectively. Y2 and Y3 are compared using consistency loss. After the weights of the student model have been updated with gradient descent in each step, the teacher model weights are updated as an exponential moving average of the student weights. Both model outputs can be used for prediction, but the teacher prediction is more likely to be correct at the end of the training. Therefore, the teacher model will be selected as the final model.
