# Supplementary material for: Explainable artificial intelligence incorporated with domain knowledge diagnosing early gastric neoplasms under white light endoscopy
Source: NPJ Digit Med. 2023 Apr 12;6:64. doi: 10.1038/s41746-023-00813-y (PMC10097818; doi:10.1038/s41746-023-00813-y)
Supplement: Supplementary file 3 — REPORTING SUMMARY [file 41746_2023_813_MOESM3_ESM.pdf]

nature portfolio

Corresponding author(s): Honggang Yu, Lianlian Wu

Last updated by author(s): Mar 21, 2023

Reporting Summary

Nature Portfolio wishes to improve the reproducibility of the work that we publish. This form provides structure for consistency and transparency in reporting. For further information on Nature Portfolio policies, see our Editorial Policies and the Editorial Policy Checklist.

Statistics

For all statistical analyses, confirm that the following items are present in the figure legend, table legend, main text, or Methods section.

n/a	Confirmed	
☐	☒	The exact sample size (*n*) for each experimental group/condition, given as a discrete number and unit of measurement
☐	☒	A statement on whether measurements were taken from distinct samples or whether the same sample was measured repeatedly
☐	☒	The statistical test(s) used AND whether they are one- or two-sided<br>*Only common tests should be described solely by name; describe more complex techniques in the Methods section.*
☒	☐	A description of all covariates tested
☒	☐	A description of any assumptions or corrections, such as tests of normality and adjustment for multiple comparisons
☐	☒	A full description of the statistical parameters including central tendency (e.g. means) or other basic estimates (e.g. regression coefficient) AND variation (e.g. standard deviation) or associated estimates of uncertainty (e.g. confidence intervals)
☐	☒	For null hypothesis testing, the test statistic (e.g. *F*, *t*, *r*) with confidence intervals, effect sizes, degrees of freedom and *P* value noted<br>*Give P values as exact values whenever suitable.*
☒	☐	For Bayesian analysis, information on the choice of priors and Markov chain Monte Carlo settings
☒	☐	For hierarchical and complex designs, identification of the appropriate level for tests and full reporting of outcomes
☐	☒	Estimates of effect sizes (e.g. Cohen's *d*, Pearson's *r*), indicating how they were calculated

Our web collection on statistics for biologists contains articles on many of the points above.

Software and code

Policy information about availability of computer code

Data collection: No software was used

Data analysis: The performance of ENDOANGEL-ED, sole DL model, and endoscopists were evaluated by accuracy, sensitivity, specificity, positive predictive value (PPV), and negative predictive value (NPV). The McNemar test was used to compare the accuracy, sensitivity, and specificity. The Chi-square test was used to compare the PPV and NPV between ENDOANGEL-ED and the sole DL model. The inter-rater agreement among the endoscopists was calculated using the Fleiss' Kappa. Performance metrics between different levels of endoscopists and ENDOANGEL-ED and the sole DL model were compared using the Mann-Whitney U test. The comparison of the acceptance and other items in the questionnaire was analyzed using Wilcoxon signed-rank Test. P-values < 0.05 were considered statistically significant.

For manuscripts utilizing custom algorithms or software that are central to the research but not yet described in published literature, software must be made available to editors and reviewers. We strongly encourage code deposition in a community repository (e.g. GitHub). See the Nature Portfolio guidelines for submitting code & software for further information.

Data

Policy information about availability of data

All manuscripts must include a data availability statement. This statement should provide the following information, where applicable:

- Accession codes, unique identifiers, or web links for publicly available datasets
- A description of any restrictions on data availability
- For clinical datasets or third party data, please ensure that the statement adheres to our policy

Individual de-identified participant data and pretraining model, software, and source code reported in this article will be shared with investigators after article publication. Data requesters could contact the corresponding author Honggang Yu (yuhonggang@whu.edu.cn) to gain access.

Human research participants

Policy information about studies involving human research participants and Sex and Gender in Research.

Reporting on sex and gender	After lesion eligibility assessment, 84 lesions (17 neoplastic, 67 non-neoplastic) with pathology results from 82 patients were included in the analysis. (50 males, 32 females)
Population characteristics	Eighty two patients were finally involved in the consecutive video test. (50 males, 32 females; 19 inpatient, 63 outpatient)
Recruitment	The performance of ENDOANGEL-ED was tested in consecutive videos of patients undertaken EGD examination from the RWHU between March 2022 and June 2022. The inclusion criteria were: 1) age ≥ 18 years; 2) sedated gastroscopy; 3) can read, understand and sign informed consent. The exclusion criteria were: 1) emergency bleeding; 2) food residues; 3) history of gastrectomy or diagnosed as remnant stomach; 4) no lesions or no pathology results. For enrolled patients, they were further selected according to the criteria for the lesions: 1) multiple lesions (more than one focal lesion in the same sight of view); 2) type I lesion, type III lesions, and ulcer; 3) the field of view was too close or too far; 4) submucosal lesions. A total of 1,441 patients who underwent EGD were consecutively enrolled. One thousand two hundred fifty-three patients who met the exclusion criteria were excluded. After lesion eligibility assessment, 84 lesions (17 neoplastic, 67 non-neoplastic) with pathology results from 82 patients were included in the analysis.
Ethics oversight	This study was approved by the Ethics Committee of RHWU. The institutional review boards exempted the informed consent for the retrospectively collected data. All the prospectively enrolled patients had signed the informed consent.

Note that full information on the approval of the study protocol must also be provided in the manuscript.

Field-specific reporting

Please select the one below that is the best fit for your research. If you are not sure, read the appropriate sections before making your selection.

☒ Life sciences ☐ Behavioural & social sciences ☐ Ecological, evolutionary & environmental sciences

For a reference copy of the document with all sections, see nature.com/documents/nr-reporting-summary-flat.pdf

Life sciences study design

All studies must disclose on these points even when the disclosure is negative.

Sample size	As for the consecutive video test, the accuracy of ENDOANGEL-ED was estimated at 80%. The sample size was calculated as 72 with an alpha of 0.05 and a power of 0.80 using the Tests for One Proportion procedure.
Data exclusions	The inclusion criteria were: 1) age ≥ 18 years; 2) sedated gastroscopy; 3) can read, understand and sign informed consent. The exclusion criteria were: 1) emergency bleeding; 2) food residues; 3) history of gastrectomy or diagnosed as remnant stomach; 4) no lesions or no pathology results. For enrolled patients, they were further selected according to the criteria for the lesions: 1) multiple lesions (more than one focal lesion in the same sight of view); 2) type I lesion, type III lesions, and ulcer; 3) the field of view was too close or too far; 4) submucosal lesions.
Replication	Five datasets were used for training, validation, and retrospective testing: 1) dataset 1, training and validation set; 2) dataset 2, internal image test set; 3) dataset 3, external image test set; 4) dataset 4, internal video test set; 5) dataset 5, external video test set. Thirteen features, including seven deep-learning features and six quantitative features, were determined by literature research and included to construct the ENDOANGEL-ED. Seven deep-learning (DL) features were extracted using deep conventional neural networks (DCNN 1-7). Feature-extraction models 1-6 were trained, validated, and tested using images in Dataset 1. The 7th feature-extraction model was previously developed. The quantitative features were extracted and analyzed based on the localized area by YOLO-v3. These quantitative features included: 1) The aspect ratio of the lesion area; 2) The spectral principal component information of the color of the lesion area; 3) The image entropy of the S-channel in the HSI color space of the lesion area; 4) The texture information of the lesion area; 5) The histogram of oriented gradient information of the lesion area; 6) The color moments of the lesion area. Seven deep-learning-based features and six quantitative features were extracted and combined and input into the fitting-diagnosis models using machine learning methods, including random forest (RF),

	Gaussian Naive Bayes (GNB), k-Nearest Neighbor (KNN), logistic regression (LR), decision tree (DT), support vector machine (SVM), and gradient boosting decision tree (GBDT). The best model was selected for constructing ENDOANGEL-ED.
Randomization	This is an single-arm observational study and the randomization is not applicable.
Blinding	This is an single-arm observational study and the blinding is not applicable.

Reporting for specific materials, systems and methods

We require information from authors about some types of materials, experimental systems and methods used in many studies. Here, indicate whether each material, system or method listed is relevant to your study. If you are not sure if a list item applies to your research, read the appropriate section before selecting a response.

Materials & experimental systems

n/a	Involved in the study	
☒	☐	Antibodies
☒	☐	Eukaryotic cell lines
☒	☐	Palaeontology and archaeology
☒	☐	Animals and other organisms
☒	☐	Clinical data
☒	☐	Dual use research of concern

Methods

n/a	Involved in the study	
☒	☐	ChIP-seq
☒	☐	Flow cytometry
☒	☐	MRI-based neuroimaging
